# Revisiting Stereocontrol through Ligand-Directed Non-Covalent Interactions in the Asymmetric Synthesis of Tetrahydrofuran Cores via Tsuji–Trost Asymmetric Allylic Alkylation

**DOI:** 10.1021/acsomega.6c03343

**Published:** 2026-06-01

**Authors:** Emanuele Casali, Lucio Toma, Alessio Porta, Giuseppe Zanoni

**Affiliations:** Department of Chemistry, University of Pavia, Viale Taramelli, 12, Pavia 27100, Italy

## Abstract

Five-membered oxygenated heterocycles, such as substituted tetrahydrofurans (THFs), are important structural motifs in bioactive compounds and can be efficiently accessed through palladium-catalyzed asymmetric allylic alkylation (AAA). In this context, the desymmetrization of mesodiols represents a powerful strategy for the stereocontrolled construction of chiral THF frameworks. Herein, we present a combined experimental and computational study aimed at elucidating the origin of an unusual diastereoselectivity inversion observed in this reaction. While ligands of the Trost family are generally considered interchangeable when sharing the same chiral information, experimental results show that switching between **(S,S)-DACH-Ph** (**L1**) and **(R,R)-ANDEN-Ph** (**L2**) leads to a reversal of the major diastereomer formed without significant loss of enantioselectivity. Density functional theory (DFT) calculations reveal that this behavior cannot be rationalized by the only classical Trost–Toste steric quadrant model. Instead, the selectivity is governed by a network of ligand-controlled noncovalent interactions, including hydrogen bonding between the ligand amide N–H, the acetate counteranion, and the substrate hydroxyl groups, which dictate the relative stability of the competing transition states. These findings provide a mechanistic framework that explains the observed disaster conversion and highlights the critical role of interaction patterns beyond steric effects, offering new predictive insights for the design of asymmetric AAA processes.

## Graphical abstract

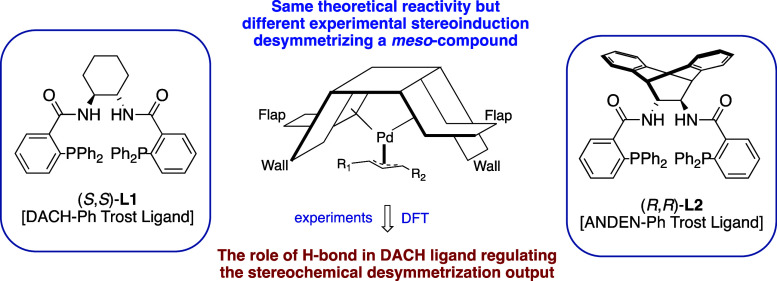

## 1 Introduction

Five-membered oxygenated heterocycles are ubiquitous motifs in natural and medicinally relevant compounds.^1^ Among them, tetrahydrofuran (THF) systems represent a particularly rich structural platform, appearing in plant-derived acetogenins, lignans, and marine macrolides as well as in physiologically relevant metabolites such as neurofurans (Figure 1).^2,3^ These THF frameworks are often central to biological activity, conferring conformational rigidity and chiral organization essential for binding and bioactivity. However, their low natural abundance and complex substitution patterns have spurred the development of efficient asymmetric synthetic routes capable of delivering these scaffolds with full stereochemical control.

**Figure 1:**
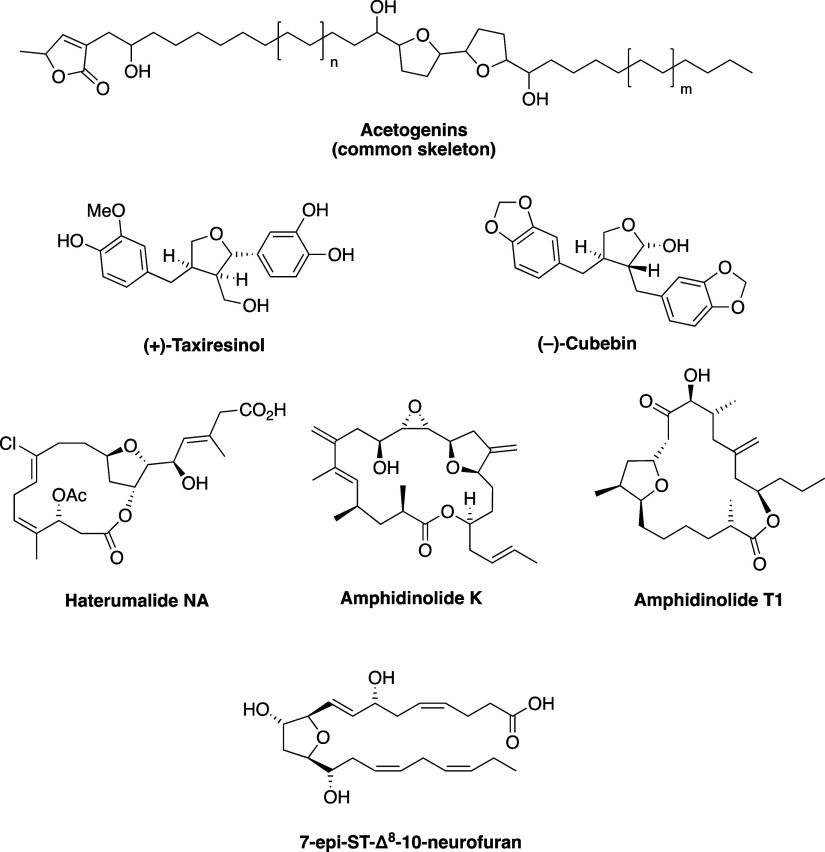
Relevant THF-containing structures from nature.

The Tsuji–Trost asymmetric allylic alkylation (AAA), employing chiral phosphine ligands coordinated to palladium(0), has become one of the most powerful catalytic strategies for enantioselective C–C and C–O bond formation.^4^ In the context of THF synthesis, the intramolecular variant of AAA enables direct desymmetrization of *meso*-allylic diols, allowing one-step conversion into enantioenriched cyclic ethers (Figure 2).

**Figure 2:**
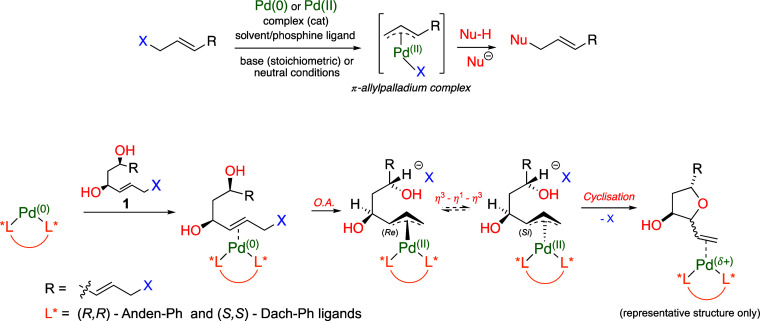
Tsuji–Trost asymmetric allylic alkylation (AAA) and the proposed mechanism for intramolecular reaction on *meso*-allylic diols.

The stereochemical outcome of this transformation is governed by the chiral environment surrounding the π-allylpalladium intermediate, which has historically been described by the Trost–Toste “cartoon model” (Figure 3).^5^

**Figure 3:**
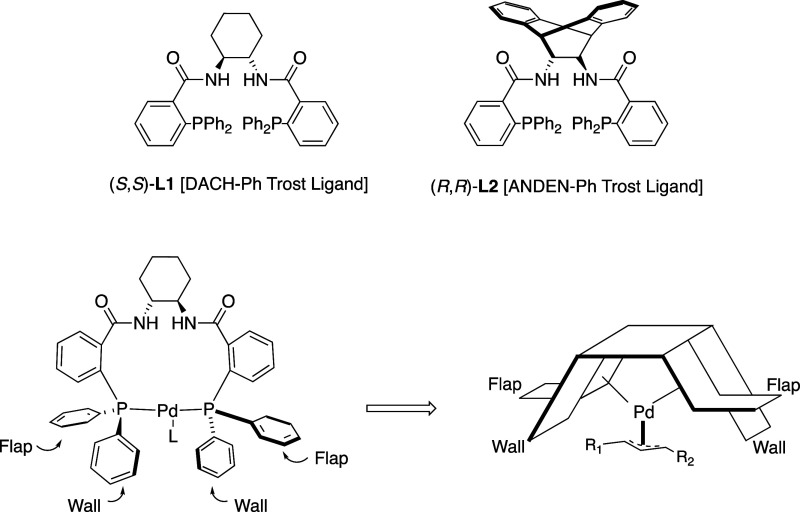
Modular Trost Ligands and the classical Trost–Toste “cartoon model”.^5^

In this classical mnemonic framework, the chiral ligand defines a set of steric quadrants around the metal center: bulky phenyl “walls” and less hindered “flaps” (see Figure 3). Enantio- and diastereoselection are then rationalized by favoring nucleophilic attack at the allyl terminus lying in the least hindered quadrant.^5,6^ Although this model successfully explains many asymmetric allylic substitutions, it assumes that the ligand’s chiral pocket exerts purely steric discrimination.^7^

Our group developed a new synthetic way to the asymmetric total synthesis of neurofurans, with the Tsuji–Trost AAA being the key step for the THF-ring closure.^3^

In that occasion, the *meso*-diol **1** was prepared as a single (*E*,*E*)-stereoisomer in 55% isolated yield, through a single-step ring-opening cross-metathesis between *cis*-cyclopent-4-ene-1,3-diol with (*Z*)-1,4-diacetoxybut-2-ene as described in Scheme 1.

**Scheme 1:**
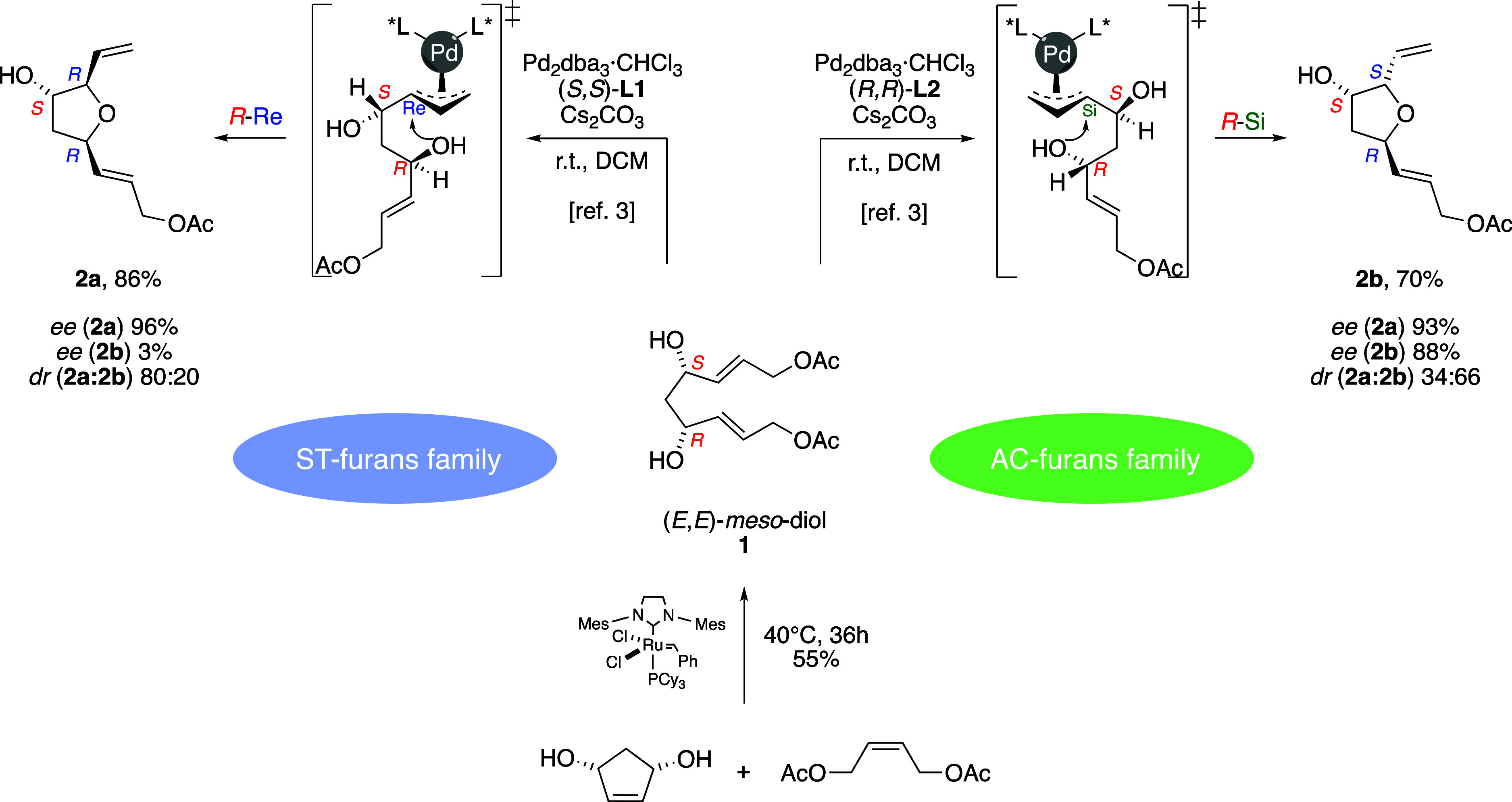
Synthesis of (*E*,*E*)-*meso*-Diol **1** through Ring-Opening Cross-Metathesis and of THF-Substituted Cores by Using Different Ligands: A Full Comprehensive Summary of the Possible Intramolecular Approaches of the Alcohol Moiety in the Presence of the Two Different Chiral Ligands^3^

The Tsuji–Trost AAA reaction forges the THF core by desymmetrizing the *meso*-compound in very high enantiomeric excess (see Scheme 1).

Under the conditions reported in Scheme 1, a diastereomeric mixture of two tetrahydrofurans ST-like **2a** and AC-like **2b** was obtained, where ST and AC stand for *syn*–*trans* and *anti*–*cis* according to the neurofuran products nomenclature, which takes into account the relative position of the allyl chains on the tetrahydrofuran ring (*syn* or *anti*) and the relationship between the hydroxyl and the olefinic adjacent geminal chain (*cis* or *trans*).^3^ When **(S**,**S)-DACH-Ph** [**(S**,**S)-L1**] was used, ST-tetrahydrofuran **2a** was obtained in 80:20 *dr* (HPLC) and 96% *e*.*e*., accompanied by the AC-diastereomer **2b**, 3% *e*.*e*., in an overall yield of 86%.

In order to increase the diastereoselectivity of the cyclization step, our group decided to change the type of ligand by using the anthracenyldiamine-derived ligand, but with the opposite absolute stereochemistry: the **(R**,**R)-ANDEN-Ph** [**(R**,**R)-L2**].^3^ Surprisingly, we observed an unanticipated inversion in the diastereomeric product distribution, which now is in favor of **2b**. This was observed by simply using the **(R**,**R)-L2** instead of **(S**,**S)-L1**, as one can see from the *dr* values 34:66 of **2a**:**2b** being the *e.e.* of **2a** and **2b**, 93% and 88%, respectively (Scheme 1).

Thus, the counterintuitive result we obtained was that the simple use of **(R**,**R)-L2** ligand does not populate the other enantiomer of the ST-furan family, as one should expect by reasoning on the stereochemistry of the ligand, but it enabled a straightforward entry to the AC-furans in good yield and high enantiomeric excess, starting from the same readily available (*E*,*E*)-*meso*-diol **1**.^3^

However, in the present system, the observed diastereo-inversion between reactions catalyzed by **(S**,**S)-L1** and **(R**,**R)-L2** ligands cannot be explained solely on steric grounds. Experimentally, both ligands produced high enantioselectivity but opposite major diastereomers, suggesting a distinct mechanism of stereocontrol, which always involves the same fragment of the molecule, but favors the intramolecular attach of the alcohol moiety to the *Si* face of the π-allylpalladium intermediate when **(R**,**R)-L2** is used, while the *Re* is the involved one in the case of the **(S**,**S)-L1** ligand (see Scheme 1). To rationalize this phenomenon, we undertook a detailed computational study aimed at disentangling the relative contributions of steric and electronic (noncovalent) factors in the AAA catalytic cycle.

Our results reveal that the key stereo-determining step is not limited to spatial quadrant accessibility but is dominated by hydrogen-bonding topologies that stabilize one transition state over another. Specifically, interactions between the ligand amide N–H, the acetate counteranion, and substrate hydroxyl groups create distinct hydrogen-bond networks in the two ligand systems, leading to opposite diastereomeric preferences.

This work, therefore, revisits the foundations of stereochemical interpretation in asymmetric palladium catalysis, providing a mechanistic link between ligand-induced H-bond networks and diastereoselectivity inversion. The DFT-based model developed here complements and extends the classical cartoon picture, offering a more nuanced view of asymmetric induction in Tsuji–Trost reactions.

## 2 Methods

The asymmetric desymmetrization of (*E*,*E*)-*meso*-diol **1** via the Tsuji–Trost asymmetric allylic alkylation (AAA) reaction was investigated to rationalize the origin of the observed diastereo-inversion upon switching between the modular Trost ligands (**S**,**S)-L1** and **(R**,**R)-L2**. The previous work from our group established that **(S**,**S)-L1** preferentially coordinates the *Si*-face of the (*S*)-allyl fragment of the substrate, whereas **(R**,**R)-L2** favors the *Re*-face to the same (*S*)-fragment.^3^ However, no clear mechanistic rationale for this selectivity inversion had been proposed.

To address this, a density functional theory (DFT) investigation was undertaken to identify the key noncovalent interactions controlling facial selectivity and stereochemical outcome. The study was inspired by Roulland and co-workers, who reported in 2012 that acetate counterions in Pd-catalyzed AAA cyclizations can act as key structural elements in counterion-directed catalysis (CDC) by mediating hydrogen-bond networks that stabilize the transition states leading to substituted tetrahydrofurans (THFs).^8^ Their work revealed that the cyclization transition state could not be located in the absence of acetate, highlighting its mechanistic indispensability.

Following this precedent, acetate was explicitly included in all of the modeled systems. It was assumed to engage in hydrogen bonding with both hydroxyl groups of substrate **1**, facilitating proton abstraction and promoting the nucleophilic attack. Population analysis was subsequently used to identify the dominant conformers contributing to the reaction coordinates.

All calculations were performed using the Gaussian 09 package^9^ at the B3LYP/6-31G­(d) level for light atoms and LanL2DZ for Pd. Conformational analysis was conducted manually to retain realistic geometries of the intact modular ligands. The allyl-acetate group on the noncoordinating fragment of meso-diol **1** was substituted with a vinyl-methyl group to reduce the complexity of the conformational search (see Figure 4). Benchmark calculations performed on the complete structure showed that this substituent does not interact with the Pd center or nearby functional groups and remains uninvolved in the transition state. Consequently, this simplification preserves the key steric and electronic features of the system while reducing the number of accessible conformations and indirectly the computational cost. The key mechanistic steps were retained, as described in Figure 2. Particularly, computational interest in terms of the final observed selectivity was placed on the steps, which establish the preference on the two allylic fragments (i.e., oxidative addition to generate the η^3^-Pd–allyl intermediate), and the one which forges the cyclized THF product. The formation of the Pd–olefin η^2^ complex and the η^3^–η^1^–η^3^ equilibration were omitted from the direct calculations. However, their influence was incorporated implicitly through a comprehensive investigation of all possible complex geometries during oxidative addition and the subsequent cyclization step.

**Figure 4:**
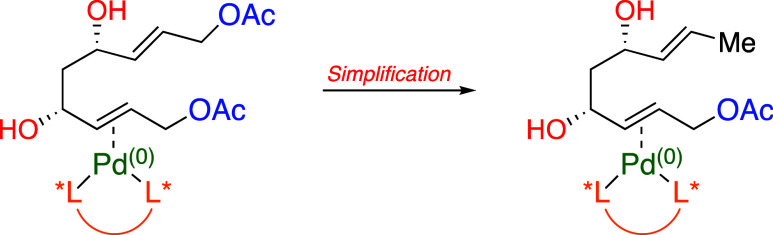
Simplification on the substrate showing the replacement of the allyl-acetate group with vinyl-methyl to reduce the number of conformational possibilities associated with the uncoordination portion of substrate **1**.

## 3 Results and Discussion

### 3.1 Mechanistic Insights with **(R**,**R)-ANDEN-Ph** [**(R**,**R)-L2**] Ligand

The **(R**,**R)-L2** ligand was selected first for its sterically demanding structure and unique conformational flexibility relative to the canonical **(S**,**S)-L1** ligand. Two principal orientations of the departing acetate group were identified: A, where the carbonyl oxygen points inward toward the allyl moiety, and B, where it points outward (Figure 5). Only the inclusion of the intramolecular H-bond between the acetate carbonyl and the unreactive hydroxyl group yielded convergent transition states, consistent with a counterion-directed catalysis (CDC) mechanism.

**Figure 5:**
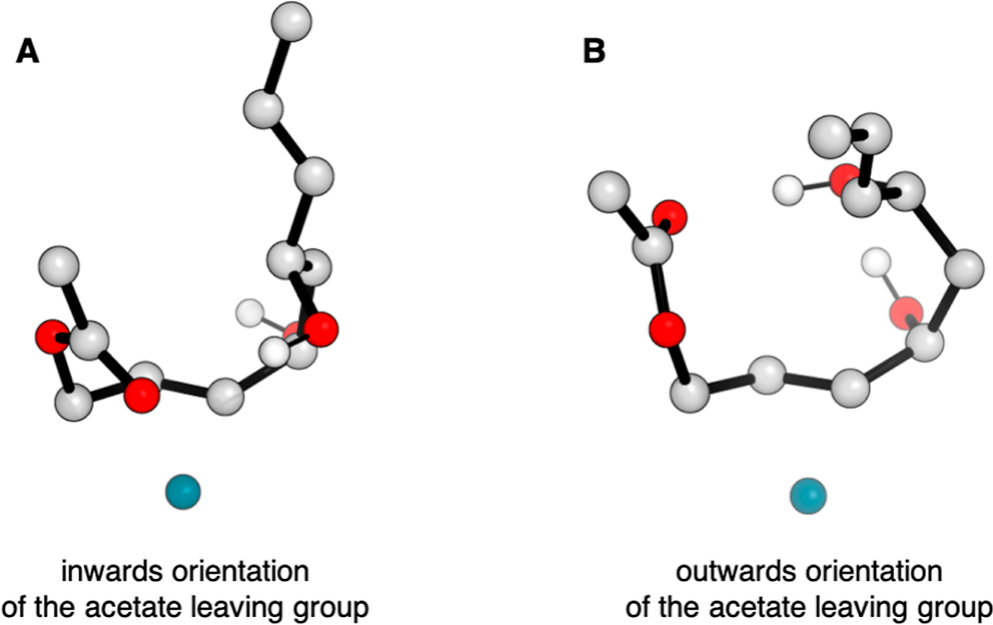
Relative orientations of the acetate leaving group “inwards” (A, left) or “outwards” (B, right).

Population analysis of all conformers revealed that oxidative addition at the (*S*)-allyl fragment (**Pro-S**) is favored over the (*R*)-one (**Pro-R**) by 3.3 kcal mol^–1^, establishing the first enantio-discriminating step (Table 1).

**Table 1:**
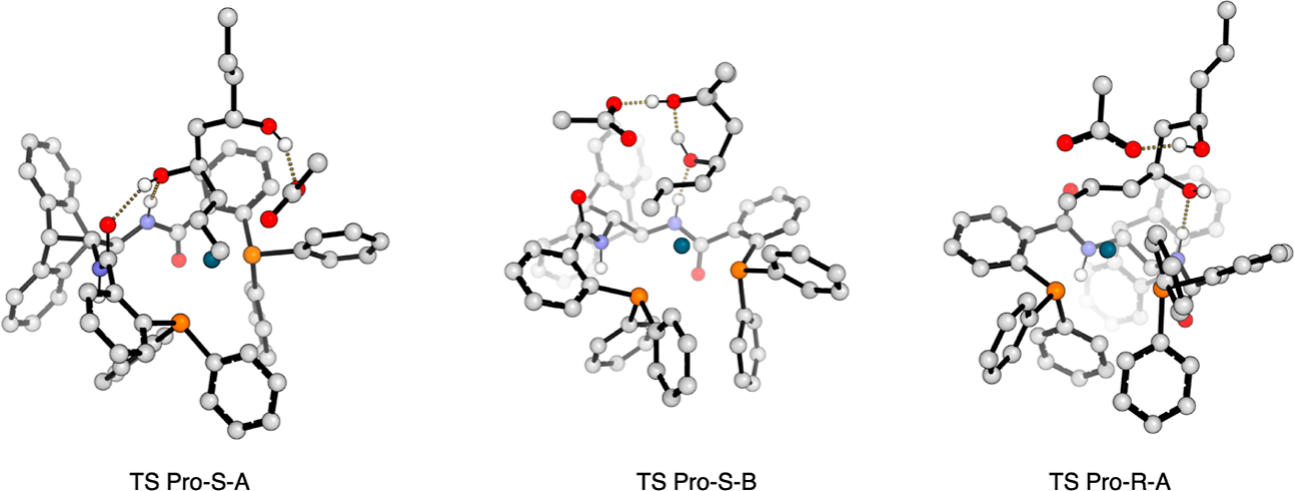
Transition State Structures for the Most Favored Pro-S-A and Pro-S-B Conformers and the Least Favored Pro-R-A One (Non-Covalent Bonds Highlighted in Yellow Dashed Lines)^a^

	*E*_rel_ (kcal mol ^–1^)	population percentage	computational	experimental
**TS Pro-S-A**	0.0	52.4	99.8	92.4
**TS Pro-S-B**	0.1	47.4		
**TS Pro-R-A**	3.3	0.2	0.2	7.6
**TS Pro-R-B**	no results	0.0		
	Sum	100.0	100.0	100.0

a DFT calculations of the four main possible conformational pathways to the oxidative addition step with the usage of (R,R)-**L2** ligand.

b Relative energy values reported in kcal/mol and population analysis for the oxidative addition step with (R,R)-**L2** ligand.

c DFT level of theory: B3LYP/6-31G­(d) and LanL2DZ.

The lowest-energy transition state (**TS Pro-S-A**) exhibited an extended H-bond network: the acetate–hydroxyl interaction (1.79 Å), the *S*-hydroxyl→ligand carbonyl donation (1.93 Å), and the amide N–H→substrate O–H interaction (2.08 Å). The combination of these three noncovalent interactions stabilizes the transition state significantly more than the competing **TS Pro-R-A** pathway, which lacks one of the intermolecular contacts (Table 1). While the **TS Pro-R-B** was not located, the **TS Pro-S-B** still shows a favorable activation energy (0.1 kcal mol^–1^) compared to the one of **TS Pro-R-A**, thus suggesting that both the A and B orientations for the acetate leaving group remain productive on the (*S*)-allyl fragment of **1**. This preference can be explained by the presence of a hydrogen-bonding network forming a continuum of interactions extending from the ligand through the substrate to the acetate leaving group. The computed structure thus confirms *Re*-face coordination of Pd to the (*S*)-allyl fragment as-hypothesized with a difference in energy greater than 3 kcal mol^–1^, which accounts for the experimentally observed high enantioselectivity. The subsequent cyclization step further amplifies this bias.

For the cyclization (Table 2), the lowest-energy pathways were **SSR-TS** and **RSR-TS**, with activation energies of 2.7 and 3.1 kcal mol^–1^, respectively, relative to the higher-energy **RRS-TS** and **SRS-TS** (11.4 and 7.5 kcal mol^–1^). The dominant transition state (**SSR-TS**) again features a tripartite H-bond motif: acetate O···H–O (1.61 Å), amide N–H···O (1.94 Å), and acetate-assisted deprotonation of the reacting hydroxyl (1.44 Å). These interactions rigidify the transition state, enforcing cyclization of the *R*–OH group onto the *Si*-face of the Pd–allyl system to yield the **SSR** product.

**Table 2:**
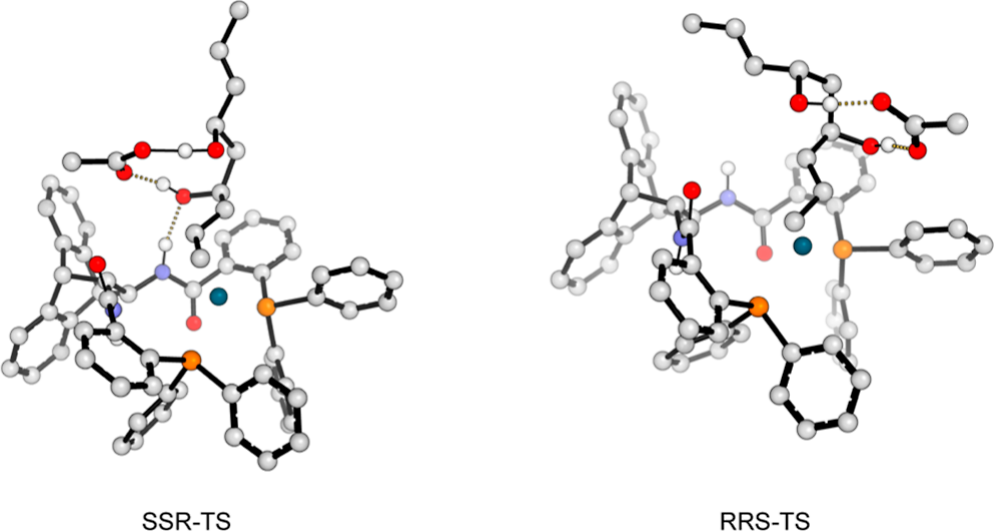
Transition State Structures for the Cyclization toward the Most Favored SSR Isomer and the Least Favored RRS One (Non-Covalent Bonds Highlighted in Yellow Dashed Lines)^a^

	*E*_rel_ (kcal mol ^–1^)	computational	experimental
**SSR-TS**	0.0	65.4	62.4
**RSR-TS**	0.4	34.6	30.0
**RRS-TS**	8.7	0.0	5.0
**SRS-TS**	4.8	0.0	2.6
	Sum	100.0	100.0

a DFT calculations of the four main possible conformational pathways to the cyclization step with the usage of **(R,R)-L2** ligand.

b Relative energy values reported in kcal/mol and population analysis for the cyclization step with **(R,R)-L2** ligand for both computational and experimental results.

c DFT level of theory: B3LYP/6-31G­(d) and LanL2DZ (only the most stable conformer for each portion is reported).

Together, these results indicate that the enantioselection occurs during oxidative addition, while the diastereoselection is determined during cyclization. The η^3^–η^1^–η^3^ equilibration plays only a permissive role, serving to interchange complexation faces rather than dictating the stereochemical outcome.

### 3.2 Mechanistic Insights with **(S**,**S)-DACH-Ph** [**(S**,**S)-L1**] Ligand

When the [**(S**,**S)-L1**] ligand was examined under identical conditions, the computed selectivity inverted relative to the experiment. DFT predicted preferential activation of the (*R*)-allyl fragment (**Pro-R**) over the (*S*)-one (**Pro-S**) by approximately 5 kcal mol^–1^ (Table 3), directly contradicting the observed experimental stereochemical outcome. The transition state (**Pro-R-A**) featured the same type of dual H-bond network as in the **(R**,**R)-L2** case, indicating that the standard model based on steric quadrant control failed to capture the experimentally relevant factors.

**Table 3:** DFT Calculations of the Four Main Possible Conformational Pathways to the Oxidative Addition Step with the Usage of **(S,S)-L1** Ligand^a^,^b^

	*E*_rel_ (kcal mol ^–1^)	population percentage	computational	experimental
**TS Pro-S-A**	4.9	0.0	0.0	78.6
**TS Pro-S-B**	no results	0.0		
**TS Pro-R-A**	0.0	95.3	100.0	21.4
**TS Pro-R-B**	1.8	4.7		
	Sum	100.0	100.0	100.0

a Relative energy values reported in kcal/mol and population analysis for the oxidative addition step with **(S,S)-L1** ligand.

b DFT level of theory: B3LYP/6-31G­(d) and LanL2DZ (only the most stable conformer for each allylic fragment is reported).

A systematic re-evaluation of computational parameters by testing a range of commonly used functionals and computational settings reported in the literature (including M06**-**2X, def2-TZVP, dispersion corrections, and implicit solvation models such as PCM/SMD, among others)^10^ failed to resolve the discrepancy, suggesting that the issue did not originate from the choice of functional, basis set, or inclusion of dispersion/solvation effects, but rather from an incomplete mechanistic description of the system. Examination of optimized structures revealed a previously unconsidered interaction between the ligand amide N–H and the acetate leaving group, an intermolecular N–H···OAc hydrogen bond capable of directly stabilizing the acetate departure.

Incorporation of this interaction yielded new transition states (**TS Pro-S-A-new** and **TS Pro-R-A-new**) that aligned quantitatively with experimental selectivities (Table 4). The **TS Pro-S-A-new** structure contained three cooperative H-bonds: (i) N–H···OAc (1.68 Å), (ii) S–OH→ligand C = O (1.84 Å), and (iii) intramolecular *R*–OH→*S*–OH (1.87 Å). The latter forms a six-membered quasi-chelate ring encompassing the two substrate oxygens, their connecting carbon skeleton, and a bridging proton, providing an additional 2–3 kcal mol^–1^ stabilization relative to the competing **TS Pro-R-A-new** geometry.

**Table 4:**
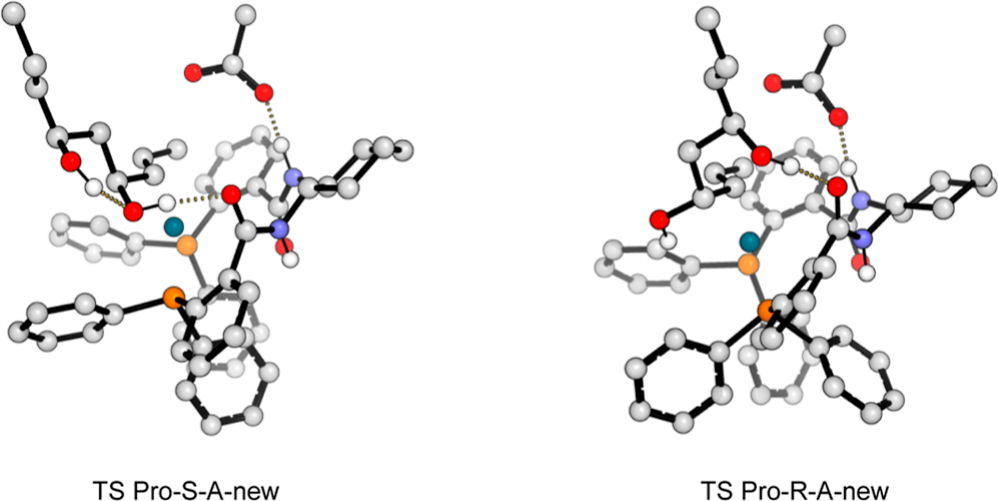
Transition State Structures for the Most Favored Pro-S-A-New Conformer and the Least Favored Pro-R-A-New One (Non-Covalent Bonds Highlighted in Yellow Dashed Lines)^a^

	*E*_rel_ (kcal mol ^–1^)	population percentage	computational	experimental
**TS Pro-S-A-new**	0.0	92.9	92.9	78.6
**TS Pro-S-B-new**	6.9	0.0		
**TS Pro-R-A-new**	1.5	7.1	7.1	21.4
**TS Pro-R-B-new**	no results	0.0		
	Sum	100.0	100.0	100.0

a Relative energy values reported in kcal/mol and population analysis for the new oxidative addition step with **(S,S)-L1** ligand.

b DFT level of theory: B3LYP/6-31G­(d) and LanL2DZ (only the most stable conformer for each portion is reported).

The topological arrangement of these noncovalent interactions inverts the energetic preference observed with **(R**,**R)-L2**. To gain more detailed insight into the energetics associated with the emergence of this novel interaction, we performed an energy decomposition analysis following the Shubin Liu protocol, as implemented in the Multiwfn software.^11,12^ By comparing the energy components of **TS Pro-S-A-new** and **TS Pro-R-A**, we identified a significant additional stabilization of the former, arising from both electrostatic interactions (9.8 kcal/mol) and steric contributions (ca. 18 kcal/mol, see Table S1 in the Supporting Information file). A more detailed analysis of the noncovalent interactions (NCIs), however, revealed that the steric contribution is largely localized within the ligand framework, particularly in the triphenylphosphine moieties, thus reflecting internal conformational effects rather than direct substrate–ligand repulsions (see Figure S2 in the Supporting Information file). In contrast, the electrostatic stabilization mainly originates from differences in hydrogen-bonding patterns between the two transition states, with a higher number of stabilizing interactions present in **TS Pro-S-A-new**.

The reaction thus proceeds through enantioselection on the (*S*)-allyl fragment, but via a distinct H-bond topology, one mediated by ligand–acetate rather than ligand–substrate interactions. This subtle difference in the spatial organization of hydrogen bonds, alongside the steric quadrant effects, is responsible for the experimentally observed diastereo-inversion. Notably, this exact N–H···OAc interaction was also independently identified as a key feature in the 2021 Tsuji–Trost AAA-based parallel kinetic resolution enabling the total synthesis of (−)-Arborisidine.^13^

### 3.3 Experimental Validation of the NH···OAc Interaction

To experimentally test the computational hypothesis, a modified ligand lacking the key N–H donor was prepared. The ligand **(S**,**S)-L1-Me** was synthesized from commercially available (1*S*,2*S*)-*N*,*N*′-dimethylcyclohexane-1,2-diamine and 2-(diphenylphosphino)­benzoic acid using EDCI/DMAP activation in dichloromethane (Figure 6).

**Figure 6:**
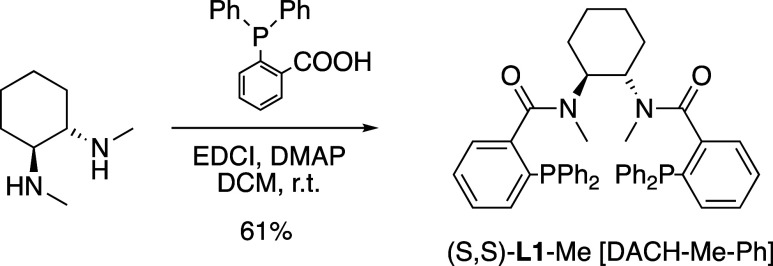
Synthesis of the ligand **(S,S)-L1-Me**.

The cyclization of (*E*,*E*)-*meso*-diol **1** using Pd_2_(dba)_3_·CHCl_3_ (3 mol %) and **(** *S*,*S* **)-L1-Me** (8 mol %) in dichloromethane proceeded smoothly to give the desired THF products in 76% isolated yield. The diastereomeric ratio shifted markedly (13:87), mirroring the **(** *R*,*R* **)-L2** behavior, while the enantiomeric excesses of both ST- and AC-furans dropped substantially (13% and 4%, respectively, Figure 7).

**Figure 7:**
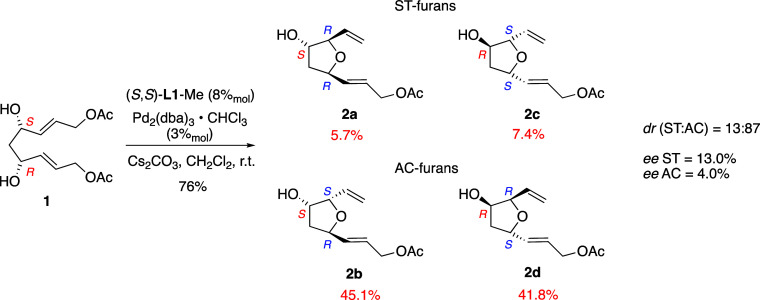
New cyclization with (*S* **,** *S*)-**L1-Me** ligand and in dichloromethane.

These results are fully consistent with computational predictions: removal of the amide N–H donor eliminates the key N–H···OAc stabilizing interaction, thereby restoring steric-dominated selectivity but diminishing overall enantio discrimination.

The experimental HPLC chromatogram confirmed the altered selectivity profile, validating that the N–H···OAc hydrogen bond is the principal determinant of stereochemical outcome in the **(** *S*,*S* **)-L1** system (see the Supporting Information for more details).

## 4 Conclusions

The collective computational and experimental findings compel a revision of the conventional Trost–Toste “cartoon model”, which attributes enantioselectivity to steric blocking by the pseudoaxial “walls” and “flaps” of the chiral pocket. While this steric quadrant model captures geometric constraints, it cannot explain the ligand-dependent reversal of diastereoselectivity observed here.

In contrast, the DFT-derived model based on noncovalent interaction topology reveals that ligand-directed hydrogen-bond networks-whose arrangement changes with ligand architecture-govern both enantio- and diastereocontrol. For **(R**,**R)-L2**, stereocontrol arises from substrate-centered interactions (ligand–hydroxyl and acetate–hydroxyl hydrogen bonds). For **(S**,**S)-L1**, control shifts to ligand-centered interactions (ligand N–H···OAc and intrasubstrate O–H···O–H contacts). These two alternative H-bond topologies invert the diastereochemical outcome without eroding enantioselectivity.

This refined mechanistic understanding transcends the purely steric paradigm of asymmetric induction. It establishes a ligand-controlled hydrogen-bond topology model as a predictive framework for designing new Trost-type ligands capable of fine-tuning selectivity through engineered noncovalent interactions rather than steric exclusion.

## Supplementary Material



## Data Availability

The data underlying this study are available in the published article and its Supporting Information.

## Notes

HPLC chromatograms, TS Cartesian coordinates, and NMR spectra (PDF)
