# Supplementary material for: Revisiting Stereocontrol through Ligand-Directed Non-Covalent Interactions in the Asymmetric Synthesis of Tetrahydrofuran Cores via Tsuji–Trost Asymmetric Allylic Alkylation
Source: ACS Omega. 2026 Jun 1;11(23):34683–90. doi: 10.1021/acsomega.6c03343 (PMC13280833; doi:10.1021/acsomega.6c03343)
Supplement: Supplementary file 1 [file ao6c03343_si_001.pdf]

Supporting information

Revisiting Stereocontrol through Ligand-Directed Non-Covalent Interactions in the Asymmetric Synthesis of Tetrahydrofuran Cores via Tsuji–Trost Asymmetric Allylic Alkylation

Emanuele Casali,* Lucio Toma, Alessio Porta, Giuseppe Zanoni*

Department of Chemistry, University of Pavia, Via Taramelli 12, Pavia 27100 (Italy)

e-mail corresponding autor: emanuele.casali@unipv.it, gz@unipv.it

General consideration and experimental procedures page S2

HPLC-Chromatograms page S3

Computational details page S4

Energy decomposition analysis and NCIs evaluation page S4

Cartesian coordinates page S6

NMR spectra (Figures S3-S7) page S22

References page S27

GENERAL CONSIDERATIONS

Unless otherwise stated, all commercial reagents were used without additional purification. Silica-gel chromatography was performed using a Merck Silicagel (SiO_2, 52–73 μm particle size, 70–230 mesh) using a proper eluent. ^{1}H NMR spectra were recorded on Bruker spectrometer (200-400 MHz). Chemical shifts were quoted in parts per million (ppm) referenced to the residual solvent peak: chloroform (7.26 ppm, ^{1}H; 77.2ppm, ^{13}C); dichloromethane (5.32 ppm ^{1}H; 54.0 ppm ^{13}C). Coupling constants, *J*, were reported in hertz (Hz). ^{13}C{^{1}H} NMR spectra were obtained on Bruker spectrometer (101 MHz), also was fully decoupled by broad band proton decoupling. ^{31}P NMR spectra was referenced to the H_3PO_4 peak. Chemical shifts were reported in ppm referenced to the residual solvent peak. Infrared (IR) spectra were acquired on Bruker Alpha ATR FT-IR Spectrometer and Perkin-Elmer Spectrum Two FT-IR ATR spectrometer. Frequencies are given in wave numbers (cm^{-1}) and only selected peaks were reported. High resolution mass spectra were obtained with High resolution QTOF mass spectrometer AB Sciex X500B by using ESI mode. Data are reported in m/z. Optical rotations were measured using a Jasco P-2000 polarimeter equipped with a temperature controller.

Synthesis of ligand (*S*,*S*)-L1-Me [(S,S)-DACH-Me-Ph Trost Ligand]:[1] In a round bottom flask, under Ar atmosphere, enantiopure (1*S*,2*S*)-*N*,*N*'-dimethylcyclohexane-1,2-diamine (1 Equiv., 100 mg, 0.70 mmol), the 2-(diphenylphosphino)benzoic acid (2 Equiv., 429 mg, 1.40 mmol), EDCI (2 Equiv., 269 mg, 1.40 mmol), DMAP (1 Equiv., 86 mg, 0.70 mmol) were added and then dissolved in dry DCM (0.2 M). The mixture was stirred for 16 hours at room temperature and was monitored by TLC (1:6:4, DCM/Hex/AcOEt). After 16 hours it was quenched removing the solvent at reduced pressure. The raw product was purified by flash chromatography [gradient of Hex/AcOEt: 7:3 then 1:1] to afford the corresponding (*S*,*S*)-**L1**-Me ligand (305 mg, 61% isolated yield). R_f = 0.20 (SiO_2, Hex/EtOAc 70:30, Vanillin, $KMnO_4$); ^{1}H NMR (400 MHz, CD_2Cl_2) δ 7.39 – 7.17 (m, 26H), 7.13 – 7.10 (m, 2H), 4.90 – 4.76 (m, 2H), 2.79 (s, 6H), 2.00 – 1.88 (m, 2H), 1.83 – 1.80 (m, 2H), 1.64 – 1.59 (m, 2H), 1.48 – 1.39 (m, 2H); ^{13}C{^{1}H} NMR (101 MHz, CD_2Cl_2) δ 170.7, 170.6, 144.3, 144.0, 137.1, 137.0, 136.6, 136.5, 134.4, 134.2, 134.1, 133.9, 133.7, 133.3, 133.1, 129.2, 128.9, 128.6, 128.5, 128.4, 128.4, 126.3, 52.1, 31.4, 29.0, 25.1; ^{31}P (162 MHz, CD_2Cl_2) δ -13.2; IR (ATR, neat, cm^{-1}) 2998, 1632, 1498, 1274; HRMS (ESI) *m/z* $[M+H]^+$ Calcd. for $C_{46}H_{45}N_2O_2P_2$ 719.2951; Found 719.2954; $[\alpha]_D^{20}$ = -39.3 (c = 0.25, $CHCl_3$). The spectroscopic data collected in our synthesis match those reported in literature.[1]

Tsuji-Trost Asymmetric-Allylic-Alkylation reaction (cyclization):[2] (*S*,*S*)-**L1**-Me (11.5 mg, 0.016 mmol, 8 mol%) and $Pd_2(dba)_3 \cdot CHCl_3$ (6.2 mg, 0.006 mmol, 3 mol %) were dissolved in dry CH_2Cl_2 (2.05 mL) under Argon atmosphere and the mixture was stirred for 45 minutes at room temperature, till the formation of the chiral complex was completed. The solution switch from form purple to orange, indicating the chiral Pd complex formation. Meanwhile in another flask, (*E*,*E*)-*meso*-diol **1**

(55.0 mg, 0.202 mmol, 1.0 Equiv.) was dissolved in dry CH_2Cl_2 (2.05 mL) under Ar, and solid Cs_2CO_3 (69.1 mg, 1.05 Equiv.) was added in one portion. Subsequently, the first solution was cannulated to the substrate-Cs_2CO_3 suspension, and the mixture was stirred until complete conversion checked by TLC (1:1, Hex/AcOEt). The reaction was then filtered and concentrated under vacuum. The oily residue was purified by liquid chromatography on silica gel [gradient of Hex/AcOEt: 8:2 > 7:3 > 1:1] to give the pure cyclized product mixture of diastereomers **2a-d** (33 mg, 76% combined yield). *d.r.* ST:AC and *e.e.*s were determined by chiral HPLC, on Chiralpak AS-H column with 80:20 Heptane:iPrOH isocratic eluent mixture flow 1.0 mL/min. R_f = 0.50 (SiO_2, Hex/EtOAc 50:50, Vanillin, $KMnO_4$); ^{1}H NMR (400 MHz, $CDCl_3$) δ 5.98 – 5.74 (m, 5H), 5.49 (dt, *J* = 17.4, 1.7 Hz, 1H), 5.41 – 5.31 (m, 2H), 5.18 (dt, *J* = 10.5, 1.4 Hz, 1H), 4.76 (dt, *J* = 10.4, 5.7 Hz, 1H), 4.70 – 4.60 (m, 1H), 4.59 – 4.55 (m, 3H), 4.52 (ddd, *J* = 5.4, 3.3, 1.6 Hz, 1H), 4.34 (s, 1H), 4.29 – 4.22 (m, 1H), 4.19 (s, 1H), 2.26 (ddd, *J* = 13.3, 6.1, 1.3 Hz, 1H), 2.14 – 1.97 (m, 7H), 1.93 – 1.81 (m, 2H); $^{13}C\{^1H\}$ NMR (101 MHz, $CDCl_3$) δ 170.7, 136.7, 134.8, 134.3, 133.3, 126.2, 125.4, 118.8, 116.7, 87.6, 83.4, 78.1, 77.7, 73.7, 64.1, 41.6, 40.5, 20.9; IR (ATR, neat, cm^{-1}) 3433, 2964, 2919, 2875, 1746, 1642, 1232, 997; HRMS (ESI) *m/z* $[M+H]^+$ Calcd. for $C_{11}H_{17}O_4$ 213.1121; Found 213.1125; $[\alpha]_D^{20}$ = 0.8 (c = 0.25, CH_2Cl_2). The spectroscopic data collected in our synthesis shows count number of proton and carbon atoms exceeding those reported in literature, due to the observed lower diastereoselectivity.[2]

HPLC-Chromatogram

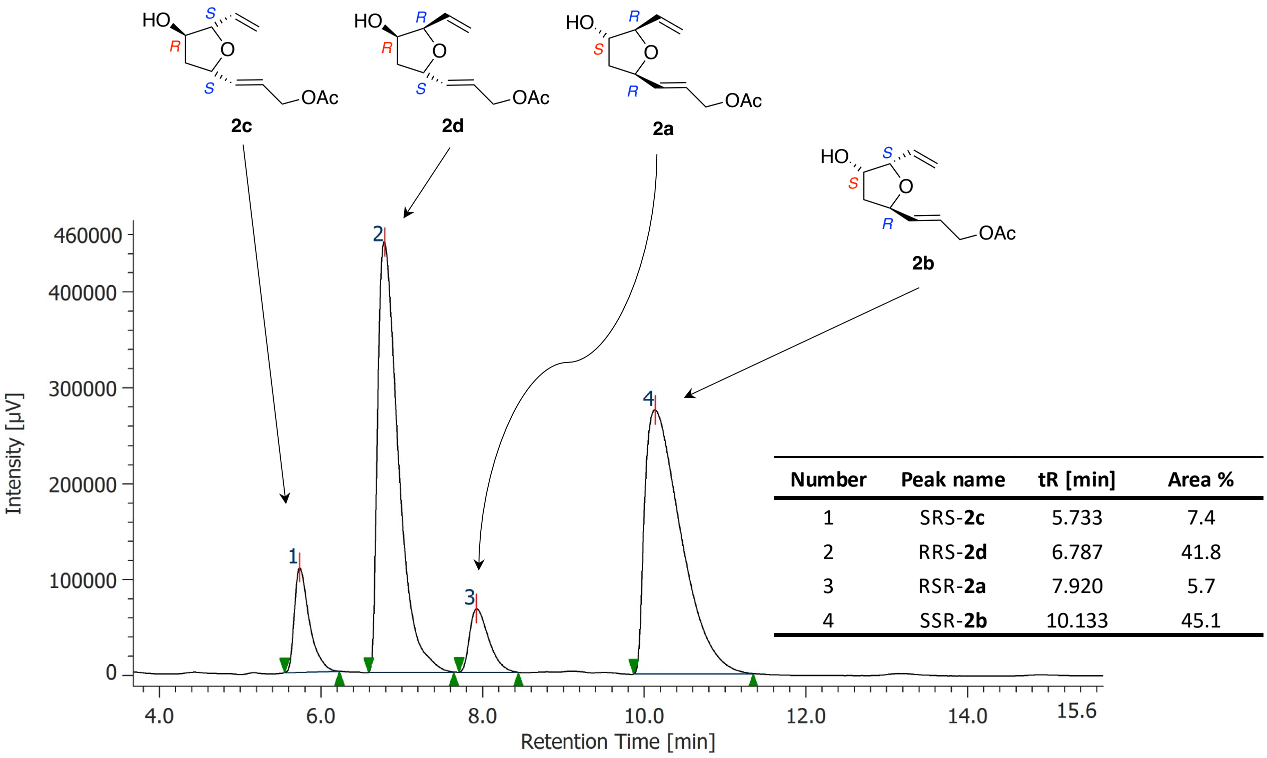

Number	Peak name	tR [min]	Area %
1	SRS-**2c**	5.733	7.4
2	RRS-**2d**	6.787	41.8
3	RSR-**2a**	7.920	5.7
4	SSR-**2b**	10.133	45.1

Figure S1: HPLC profile for the (S,S)-**L1**-Me promoted cyclization and the corresponding integrations. HPLC conditions: Chiralpak AS-H, heptane/*i*-PrOH 80:20, 1.0 mL/min, DAD detection, 212 nm. The peak attribution was performed by following the same HPLC conditions described in literature.[1]

Computational details

All structures were optimized with the Gaussian 09 program package,[3] using the B3LYP[4] functional at the 6-31G(d) level for all the atoms,[4] but for Palladium the effective core potential LanL2DZ was used.[6] All the optimizations were performed in vacuo and the existence of the transition state was validated through frequency analysis and intrinsic reaction pathway calculation.[7] Conformational sampling was carried out through a systematic, manual exploration of all relevant degrees of freedom. In particular, were considered: (i) the different orientations of the substrate with respect to the Pd center, (ii) the possible orientations of the acetate leaving group relative to the substrate (notably the A and B arrangements), (iii) the relative orientations between the two hydroxyl groups in the substrate and between the cyclisation involved hydroxyl group and the acetate leaving group, and (iv) the relative orientations of the phenyl substituents on the phosphine ligands. From this extensive exploration, only the lowest-energy and most relevant conformers were retained for further analysis.

All data reported below, are referred to this level of theory and the discussions are based on the values of (E) and (E_{rel}) relative energies in kcal/mol. Boltzmann population analysis was performed at the reaction specified temperature.

Energy decomposition analysis and NCIs evaluation

To gain more detailed insight into the energetics associated with the emergence of this novel interaction, we performed an energy decomposition analysis following the Shubin Liu protocol, as implemented in the Multiwfn software using the optimized structures of **TS Pro-S-A-new** and **TS Pro-R-A**.[8,9]

In Table S1 the direct comparison between the energies of the two transition states is reported.

	TS-Pro-R-A	TS-Pro-S-A-new	Δ (kcal/mol)
**Electronic kinetic energy (E_K – a.u.)**	3400.966704	3400.984774	11.3
**Weizsacker kinetic energy (K_W – a.u.)**	5206.173624	5206.144755	-18.1
**Interelectronic Coulomb repulsion energy (E_J – a.u.)**	13975.615198	13845.068036	-81919.6
**Internuclear Coulomb repulsion energy (E_{nuc} – a.u.)**	12276.564613	12145.933390	-81972.4
**Nuclear-electronic Coulomb attraction energy (E_V – a.u.)**	-32708.970271	-32447.807493	163882.3
**Energy without electronic correlation (E_K+E_V+E_J+E_{nuc} – a.u.)**	-3055.823756	-3055.821293	1.5
**Exchange correlation energy (E_x – a.u.)**	-423.260003	-423.267464	-4.7
**Coulomb correlation energy (E_c – a.u.)**	-21.989733	-21.989148	0.4
**Pauli kinetic energy (E_K-K_W – a.u.)**	-1805.206920	-1805.159981	29.5
**EDA-SBL energy decomposition terms**			
**E_{steric} (a.u.)**	5206.173624	5206.144755	-18.1
**$E_{electrostatic}$ (a.u.)**	-6456.790460	-6456.806067	-9.8
**$E_{quantum}$ (a.u.)**	-2250.456656	-2250.416593	25.1
**E_{total} (a.u.)**	-3501.073492	-3501.077905	-2.8

Table S1: Energy decomposition analysis following the Shubin Liu protocol (EDA-SBL) using Multiwfn.[8,9] Difference between **TS Pro-S-A-new** and **TS Pro-R-A** is reported in kcal/mol.

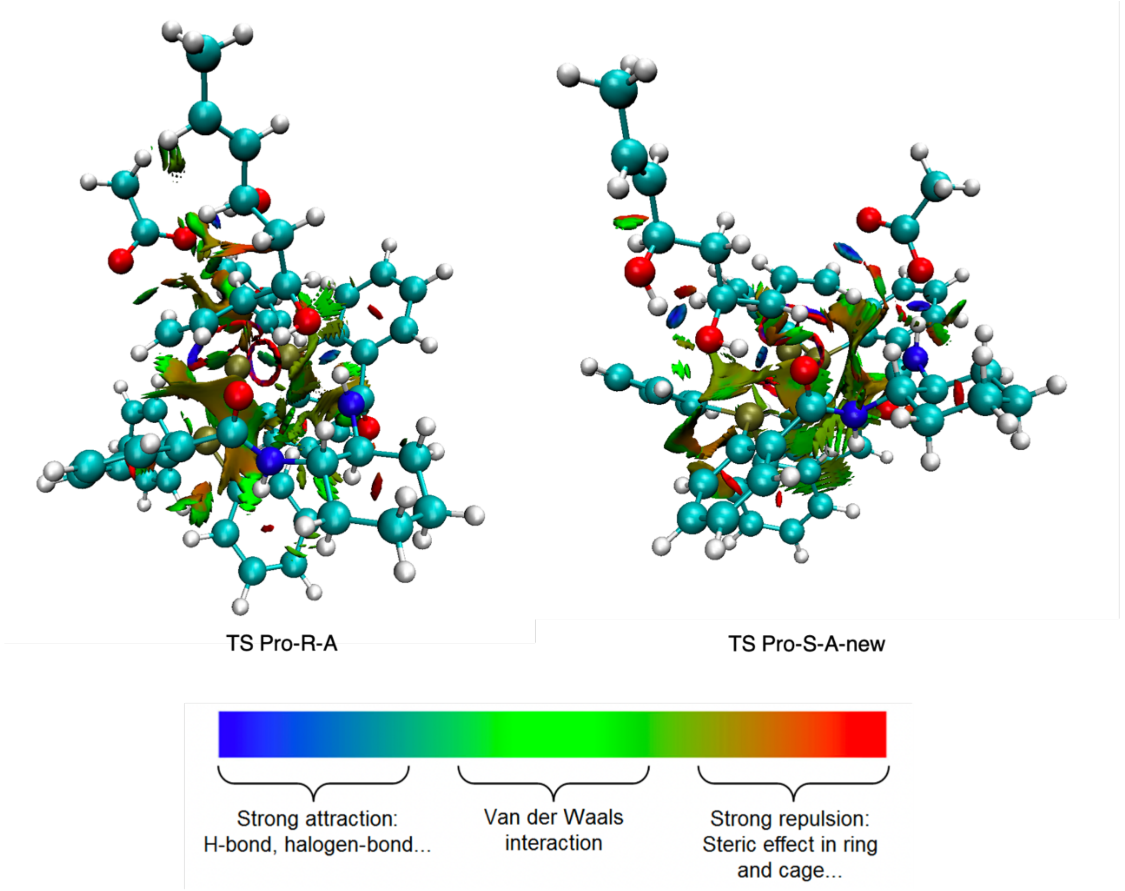

Figure S2: Non-covalent interaction (NCI) isosurfaces for **TS-Pro-S-A-new** and **TS Pro-R-A**. Steric contributions (brown) are mainly localized within the ligand framework, particularly the triphenylphosphine moieties, indicating internal conformational strain rather than direct substrate-ligand repulsions. Electrostatic interactions (blue) highlight differences in hydrogen-bonding patterns between the two transition states.

(R,R)-ANDEN-Ph [(R,R)-L2] Trost Ligand investigation

TS Pro-S-A
b3lyp/6-31g(d)&LanL2DZ,
el. energy = -3883.333112 a.u.
im. frequency -102.56

C	-1.75852800	2.65792500	0.10184600
C	-2.26326200	1.36585200	-0.48973100
O	-0.34659400	2.87052100	-0.08135300
C	-2.55211800	3.87882600	-0.44254400
C	-1.94438500	0.95295300	-1.80958900
H	-3.24782600	1.07889400	-0.13871300
C	-2.47312000	-0.25383800	-2.26330500
H	-1.21112100	1.47302500	-2.41880300
H	-3.30360200	-0.72439300	-1.75680800
H	-2.17839400	-0.66095700	-3.22467600
H	-2.10730800	4.18246400	-1.40010500
C	-4.06077700	3.63534800	-0.67172100
H	-2.39758000	4.71108000	0.25560300
O	-4.62906800	2.96458300	0.45159900
H	-5.09293000	2.16914800	0.08702700
H	-0.11451000	2.77651900	-1.02503400
H	-1.89705200	2.61507200	1.18315400
H	-4.16533400	3.00433900	-1.56398100
C	-6.99439400	1.21118700	-2.42370400
H	-7.23278600	1.07647200	-3.48157100
H	-7.76986200	0.74326900	-1.80653700
H	-6.99923500	2.28178100	-2.18385100
C	-5.62028300	0.61272300	-2.07854800
O	-4.93625400	0.14245800	-3.01914800
O	-5.28658100	0.65293800	-0.84446200
N	1.75407700	1.55628100	1.46330200
C	2.96546900	1.20698600	0.73604400
C	2.90169400	1.73916200	-0.71975700
N	2.48350300	0.69943300	-1.65382100
C	1.31990100	0.76155500	2.47637900
C	1.49674200	0.90894400	-2.56095200
O	0.90879400	1.99574100	-2.65730400
O	1.94647400	-0.22248000	2.87691900
C	0.05042700	1.22496100	3.15236000
C	1.18845900	-0.20555000	-3.53813700
C	-1.14935500	0.47554300	3.15572400
C	-2.23912900	0.97461000	3.88613500
C	-2.14937000	2.16932800	4.60366000
C	-0.96567900	2.90207600	4.59251200
C	0.12535900	2.42840200	3.86356100
C	1.27877200	0.19021300	-4.88085900
C	1.01430300	-0.69755500	-5.92052000
C	0.63056200	-2.00198900	-5.61998600
C	0.50838100	-2.40202300	-4.28862400
C	0.78154900	-1.52819000	-3.22203100
P	-1.38083300	-0.99669500	2.04420300
P	0.45431400	-2.10024600	-1.47045600
C	-3.13335700	-1.51835900	2.35845400
C	-0.49522200	-2.40988100	2.84631100
C	2.15718300	-2.49299400	-0.86684300
C	-0.30677100	-3.77361100	-1.68311800
C	0.44482100	-4.95775400	-1.74941100
C	-0.19210800	-6.19331600	-1.88370900
C	-1.58468900	-6.26376800	-1.95540700
C	-2.34226900	-5.09262000	-1.88614600
C	-1.70860800	-3.85772300	-1.74346300
C	0.17105200	-2.31794900	4.07484500
C	0.76252500	-3.45075800	4.64029500
C	0.69274100	-4.68367800	3.99043400
C	0.02728000	-4.78383200	2.76594800
C	-0.56115300	-3.65469600	2.19795300
C	-3.46972400	-2.25575000	3.51009600
C	-4.79385400	-2.61429800	3.75861500
C	-5.80469800	-2.24440500	2.86571800
C	-5.48169500	-1.51658400	1.72184300
C	-4.15420600	-1.16184500	1.46719200
C	2.40230500	-2.50217400	0.51410400
C	3.66879400	-2.82448400	1.00624300
C	4.70783700	-3.13138800	0.12691900
C	4.47825700	-3.11603200	-1.25096500
C	3.21127200	-2.80176700	-1.74756700
H	1.07608800	2.19237000	1.03483400
H	3.04046700	0.11717700	0.73021000
H	2.14800200	2.52001700	-0.80140500
H	2.98295700	-0.18215100	-1.65712500
H	-3.17885900	0.43559200	3.88408700
H	-3.01340700	2.52677400	5.15646400
H	-0.89034000	3.83904200	5.13715400
H	1.05270200	2.99379400	3.83984800
H	1.55306300	1.21793300	-5.09724800
H	1.09594200	-0.36708900	-6.95189600
H	0.41129800	-2.70959400	-6.41471700
H	0.18126100	-3.41353000	-4.07692500
H	1.52765900	-4.92016000	-1.69047800
H	0.40323600	-7.10137600	-1.93008700
H	-2.07758800	-7.22664800	-2.05835500
H	-3.42666200	-5.13672000	-1.93333200
H	-2.30622400	-2.95419700	-1.67087400
H	0.24780200	-1.36417900	4.58230400
H	1.27961500	-3.36433600	5.59229700
H	1.15374900	-5.56197700	4.43470600
H	-0.03336200	-5.73829500	2.25009600
H	-1.07865100	-3.74430400	1.24661100
H	-2.69870400	-2.55206600	4.21397300
H	-5.03528500	-3.18411900	4.65235600
H	-6.83610900	-2.52336100	3.06573900
H	-6.23980200	-1.19829100	1.01307700
H	-3.95928900	-0.59884900	0.56459000
H	1.61794600	-2.23805400	1.21186000
H	3.83616900	-2.80956800	2.07877700
H	5.69665500	-3.36648300	0.50977400
H	5.28504700	-3.34482200	-1.94190300
H	3.04407100	-2.79822000	-2.82090000
Pd	-1.05249200	-0.50767400	-0.34963000
C	4.28236900	1.73408100	1.41689900
H	4.29001500	1.44166900	2.46938800
C	4.27452200	2.40663100	-1.08873000
H	4.27674400	2.70026800	-2.14205000
C	6.34146000	0.91330900	-1.61029200
C	7.32673800	0.05029700	-1.11491700

C	7.35118100	-0.28495400	0.23999700
C	6.38725900	0.23245200	1.11432600
C	5.41174000	1.09627000	0.62524300
C	5.39086000	1.43922000	-0.73903200
H	6.32696900	1.18209000	-2.66423200
H	8.07919100	-0.35267000	-1.78770300
H	8.12419900	-0.94771200	0.62034300
H	6.40319200	-0.03231000	2.16883100
C	4.42800700	4.94151200	-0.51787300
C	4.47681000	5.92489900	0.47757700
C	4.46152400	5.56344700	1.82571700
C	4.39820700	4.21431300	2.19492300
C	4.35721900	3.23475400	1.20677700
C	4.37481700	3.60029100	-0.15054300
H	4.43211200	5.22346500	-1.56820500
H	4.52460400	6.97365000	0.19737100
H	4.49854700	6.33188000	2.59321400
H	4.38612000	3.93234600	3.24524000
C	-4.78577800	4.93551600	-0.91886600
C	-5.29919400	5.31604000	-2.09161700
H	-4.88423800	5.58108700	-0.04381600
H	-5.19890200	4.64695300	-2.94892100
C	-6.02340400	6.60831800	-2.34544100
H	-5.51670200	7.20715000	-3.11524600
H	-7.04358600	6.42771300	-2.71118700
H	-6.09257700	7.21537800	-1.43603700

TS Pro-S-B

b3lyp/6-31g(d)&LanL2DZ,
el. energy = -3883.333018 a.u.
im. frequency -17.15

C	-0.01733500	3.23723200	-1.23818500
C	0.63427400	2.70035600	0.01916200
O	-1.36098900	2.78469000	-1.38011900
C	0.03538200	4.78321100	-1.34472800
C	-0.11485200	2.21677100	1.09596200
H	1.65191200	3.03864500	0.20284100
C	0.51271200	1.65386300	2.21456800
H	-1.19152300	2.13250300	1.00792800
H	1.52438000	1.94960200	2.48246100
H	-0.08721900	1.23635000	3.00985200
H	-0.44422100	5.01720900	-2.30379500
C	-0.69285600	5.55829900	-0.21194300
H	1.07670800	5.12060500	-1.41155200
O	-1.90266200	4.86479900	0.09374800
H	-2.01760300	4.72174500	1.10434500
H	-1.88310600	3.44449800	-0.83966500
H	0.53819300	2.85368400	-2.09779700
H	-0.94690900	6.54497400	-0.63485100
C	-2.35904500	3.58016600	4.81872600
H	-1.68129800	3.64746600	5.67507900
H	-3.23785400	4.21229200	4.97836200
H	-2.70616500	2.54274400	4.72825800
C	-1.64119800	3.96070900	3.51398000
O	-0.38644800	3.82773700	3.47882400
O	-2.39833300	4.33970900	2.56903600
N	-1.57729800	-0.02552300	-2.09065300
C	-2.46014600	-0.97331700	-1.42178200
C	-2.90911200	-0.44553200	-0.03066900
N	-2.16652800	-1.09783800	1.04840100
C	-0.52975400	-0.46610800	-2.82517700
C	-2.09396000	-0.50806300	2.27643500
O	-2.65685900	0.55984400	2.51322000
O	-0.27169000	-1.66149200	-3.00841200
C	0.31551400	0.59876200	-3.48352200
C	-1.31701500	-1.19352800	3.37976600
C	1.67305000	0.80806900	-3.14351300
C	2.40056200	1.75867400	-3.87565700
C	1.81412400	2.47345200	-4.92275500
C	0.47577600	2.26443300	-5.24640900
C	-0.26716700	1.33168300	-4.52269700
C	-1.98862100	-1.21003500	4.61198400
C	-1.39475100	-1.72838800	5.75821200
C	-0.08987900	-2.21237600	5.69060600
C	0.60230600	-2.17683400	4.48048600
C	0.01104400	-1.68054400	3.30485700
P	2.39812200	-0.00316400	-1.63523900
P	1.00601500	-1.67972100	1.72670500
C	4.03866200	0.83129400	-1.39647300
C	2.98957100	-1.66141400	-2.21126300
C	0.41621200	-3.25423600	0.95145400
C	2.71725900	-2.07162500	2.30899500
C	3.28063000	-3.35447500	2.23928400
C	4.59085600	-3.57607000	2.67273800
C	5.35117800	-2.52420300	3.18510900
C	4.79888100	-1.24263100	3.25883500
C	3.49599600	-1.01689400	2.81645400
C	2.70004100	-2.18417100	-3.47819000
C	3.20355100	-3.43205200	-3.85559300
C	3.99851800	-4.16889600	-2.97753800
C	4.29104200	-3.65322000	-1.71233400
C	3.79042700	-2.40937200	-1.33137400
C	5.11109200	0.60112500	-2.27751400
C	6.34052300	1.22890700	-2.08054000
C	6.52554500	2.08851900	-0.99432100
C	5.47613000	2.31159200	-0.10335800
C	4.24368800	1.68436700	-0.30314700
C	0.38775300	-3.37244200	-0.44468400
C	-0.04534300	-4.55438700	-1.05057700
C	-0.45123000	-5.63479700	-0.26795800
C	-0.43233200	-5.52757800	1.12563400
C	-0.00770600	-4.34604300	1.73373500
H	-1.65386300	0.97961100	-1.89220800
H	-1.88935200	-1.89761800	-1.31185900
H	-2.69033400	0.62003900	0.05009300
H	-1.85975700	-2.05471900	0.92983300
H	3.43353600	1.96230800	-3.61948000
H	2.40546000	3.20113500	-5.47155200
H	0.00737800	2.82781700	-6.04832000
H	-1.31486800	1.16830200	-4.75741600
H	-2.98560500	-0.78456300	4.65429400
H	-1.94014800	-1.73580500	6.69741300

H	0.39906800	-2.60736000	6.57689700
H	1.62366100	-2.53894700	4.45438200
H	2.70174000	-4.18242000	1.84422600
H	5.01385700	-4.57530100	2.61108800
H	6.36886300	-2.69971500	3.52273600
H	5.38371000	-0.41709700	3.65488000
H	3.07796600	-0.01556000	2.86827400
H	2.06503300	-1.63565400	-4.16234700
H	2.96982000	-3.82523900	-4.84138200
H	4.38810800	-5.13867500	-3.27553600
H	4.90870500	-4.21738000	-1.01874900
H	4.03429900	-2.01861600	-0.34770700
H	4.98717100	-0.07759700	-3.11589300
H	7.15666800	1.04133100	-2.77307900
H	7.48524800	2.57430100	-0.84061200
H	5.61267700	2.96948000	0.75057200
H	3.43912900	1.84512400	0.40619400
H	0.67618300	-2.54311300	-1.07526500
H	-0.07152300	-4.60746200	-2.13441500
H	-0.79332000	-6.55304700	-0.73766400
H	-0.75754200	-6.36104700	1.74248000
H	-0.01154900	-4.27363800	2.81656000
Pd	1.04425000	0.37850300	0.42765100
C	-3.73430500	-1.32788700	-2.27375500
H	-3.42700500	-1.64401700	-3.27415500
C	-4.46830300	-0.59448900	0.10325800
H	-4.77866200	-0.27471400	1.09975800
C	-5.45105600	-2.94220600	0.64149300
C	-5.70443400	-4.24507300	0.19538900
C	-5.32137800	-4.63625000	-1.08888600
C	-4.67883800	-3.72981200	-1.94104700
C	-4.43591100	-2.43135400	-1.50179900
C	-4.82214300	-2.03617900	-0.20844000
H	-5.75133300	-2.63577200	1.64086200
H	-6.20418400	-4.95275300	0.85166800
H	-5.52363400	-5.64790800	-1.43089600
H	-4.37678800	-4.03489300	-2.94035700
C	-5.83720300	1.41324000	-0.82317900
C	-6.25084900	2.13946400	-1.94623600
C	-5.85853900	1.74377900	-3.22626300
C	-5.04246800	0.61949500	-3.39904300
C	-4.63465000	-0.10666100	-2.28350900
C	-5.03802000	0.28663700	-0.99593600
H	-6.13055200	1.72970600	0.17445200
H	-6.87537100	3.01911900	-1.81782900
H	-6.18327300	2.31360000	-4.09285000
H	-4.73223700	0.31330100	-4.39580800
C	0.16359900	5.78706800	1.01271300
C	0.87163200	6.90227300	1.21717200
H	0.15400700	5.00834900	1.77875900
H	0.83786000	7.70810400	0.47829300
C	1.71996900	7.15055200	2.43405200
H	1.41753800	8.07492300	2.94578900
H	1.62835100	6.32359600	3.14579300
H	2.78202900	7.27239700	2.17369500

TS Pro-R-A
b3lyp/6-31g(d)&LanL2DZ,
el. energy = -3883.327896 a.u.
im. frequency -119.54

C	-1.77136500	2.45024400	-0.84989100
C	-2.05388500	0.98506400	-1.09808800
C	-2.90224800	3.31255200	-1.48363500
C	-1.56904200	0.37246300	-2.27149800
H	-2.99867500	0.62371200	-0.71759600
C	-1.92262600	-0.95236100	-2.53622500
H	-0.85715900	0.88402600	-2.91371100
H	-2.74327100	-1.42946000	-2.02105600
H	-1.49604900	-1.47551300	-3.38577600
H	-3.21276600	2.85157900	-2.42914700
C	-4.12699900	3.50652300	-0.55595400
H	-2.50171800	4.30418100	-1.72926500
O	-4.46382300	2.31118000	0.15193300
H	-4.79056800	1.63182800	-0.49446000
C	-6.27662200	0.58639200	-3.53381500
H	-6.58225900	-0.02764700	-4.38555900
H	-7.11041300	0.70964000	-2.83605100
H	-6.00851400	1.57884500	-3.92082400
C	-5.05443100	-0.02746800	-2.82837900
O	-4.27544600	-0.70913500	-3.53967600
O	-4.92212300	0.22573900	-1.58477500
N	1.04528300	1.86873800	1.55622100
C	2.35732200	1.80349700	0.92951600
C	2.28341200	2.07108700	-0.59846300
N	2.35760200	0.82869500	-1.36318000
C	0.74029500	1.04849000	2.59420700
C	1.83283400	0.76324900	-2.61928700
O	1.28724200	1.74101500	-3.13861000
O	1.54199300	0.25328500	3.09093300
C	-0.65226800	1.21634300	3.15471300
C	1.96147000	-0.52509900	-3.40450500
C	-1.66645100	0.25073400	2.97396300
C	-2.91163400	0.47391100	3.58203500
C	-3.14654900	1.61155800	4.35682200
C	-2.13816800	2.55646000	4.53281100
C	-0.89709100	2.35573500	3.92847800
C	2.29783300	-0.31489900	-4.75111600
C	2.42577500	-1.37175900	-5.64718900
C	2.19039400	-2.67092500	-5.20413200
C	1.82994500	-2.89629700	-3.87548900
C	1.71117400	-1.84603000	-2.94806800
P	-1.41050500	-1.20224700	1.84204200
P	1.14589200	-2.23844200	-1.21000000
C	-3.07943000	-2.01608300	1.81522100
C	-0.46371100	-2.42876500	2.85789000
C	2.75564200	-2.24246900	-0.29447700
C	0.69103500	-4.03039100	-1.29080300
C	1.60403500	-5.07124700	-1.05879400
C	1.18515700	-6.40297700	-1.10505200
C	-0.14705100	-6.71340600	-1.38529300
C	-1.06463000	-5.68549000	-1.61331900
C	-0.64999400	-4.35435700	-1.55822000

C	-0.01037400	-2.17683500	4.15923500
C	0.67361800	-3.16614600	4.87100700
C	0.90956000	-4.41545200	4.29650700
C	0.45811400	-4.67609100	3.00018200
C	-0.22091600	-3.68950600	2.28676100
C	-3.46644200	-2.92690600	2.81605000
C	-4.73795700	-3.50069000	2.79832100
C	-5.64618600	-3.17349600	1.78754400
C	-5.27507500	-2.26692500	0.79526500
C	-4.00015700	-1.69681900	0.80654200
C	2.75823100	-1.97748900	1.08365400
C	3.95405700	-1.98541400	1.80492700
C	5.16132100	-2.25706800	1.16015500
C	5.17240900	-2.51614200	-0.21267200
C	3.97977300	-2.50677400	-0.93812300
H	0.27980700	2.41165800	1.15328100
H	2.73293800	0.79569200	1.11830000
H	1.32448700	2.52259800	-0.85440200
H	2.95671000	0.08371800	-1.02951900
H	-3.71418700	-0.24033900	3.44093500
H	-4.12253400	1.75756500	4.81084400
H	-2.31556700	3.44876200	5.12665600
H	-0.10666900	3.09157400	4.04827200
H	2.44287200	0.70736900	-5.08376000
H	2.69390100	-1.17858000	-6.68189400
H	2.27452100	-3.51178100	-5.88718200
H	1.63042800	-3.91288000	-3.55684800
H	2.64186400	-4.84586400	-0.83584200
H	1.90292300	-7.19774600	-0.91963400
H	-0.47019900	-7.75029900	-1.41858000
H	-2.10554400	-5.91550600	-1.82252900
H	-1.37516500	-3.56023100	-1.70796900
H	-0.17284100	-1.20781900	4.61471000
H	1.02147700	-2.95442900	5.87866600
H	1.44159500	-5.18168000	4.85433500
H	0.63625200	-5.64402100	2.53968400
H	-0.57247900	-3.90629500	1.28253000
H	-2.77716900	-3.18856600	3.61160800
H	-5.01839600	-4.20358000	3.57875000
H	-6.63654900	-3.62150300	1.77897300
H	-5.95958600	-1.97855100	0.00346800
H	-3.76818200	-0.99609500	0.01578900
H	1.83668000	-1.74158500	1.60003900
H	3.92852200	-1.75825300	2.86610900
H	6.09262500	-2.25443600	1.71985600
H	6.11003400	-2.71851600	-0.72332600
H	4.00247400	-2.70240600	-2.00595200
Pd	-0.67770100	-0.74645600	-0.52294300
C	3.39748200	2.79336900	1.57372400
H	3.40663300	2.65425100	2.65762300
C	3.39169100	3.11301200	-0.99895900
H	3.38297900	3.25478300	-2.08187600
C	5.85029900	2.28899000	-1.21678100
C	6.99956700	1.82389700	-0.56590200
C	7.00785400	1.66707700	0.82128900
C	5.86726100	1.97223500	1.57395800
C	4.72765000	2.44452500	0.92882300
C	4.71870700	2.60278400	-0.46847700
H	5.84323800	2.41213900	-2.29738700
H	7.88874800	1.58684700	-1.14418300
H	7.90403800	1.30880300	1.32109400
H	5.87133400	1.84537200	2.65400500
C	2.69052100	5.60967600	-0.79532000
C	2.35023500	6.67318500	0.04919400
C	2.34195100	6.49825700	1.43418300
C	2.67434500	5.25766600	1.99149900
C	3.02099800	4.20152000	1.15352200
C	3.03099900	4.37904800	-0.24093600
H	2.68993900	5.74282800	-1.87452300
H	2.08984300	7.63819300	-0.37715100
H	2.07580700	7.32783800	2.08375400
H	2.66744900	5.12071100	3.07045200
H	-3.84383700	4.22418500	0.22270600
O	-1.59713400	2.76391800	0.53412900
H	-2.41687400	2.47169300	0.97775700
H	-0.82560000	2.72149300	-1.33058400
C	-5.30401200	4.05795600	-1.31903800
C	-5.80578200	5.28527900	-1.16250400
H	-5.75027900	3.37555900	-2.04519200
H	-5.34684900	5.94826300	-0.42573400
C	-6.97070300	5.85171700	-1.92363100
H	-7.37979100	5.12359500	-2.63214500
H	-7.77876800	6.15619500	-1.24432700
H	-6.68108400	6.74905800	-2.48800600

SSR-TS

b3lyp/6-31g(d)&LanL2DZ,

el. energy = -3883.349415 a.u.

im. frequency -150.58

C	2.45122300	-2.57526400	-0.27320300
C	1.63511400	-2.46708900	0.99728700
O	3.05815500	-1.36352500	-0.66052100
C	3.45250400	-3.73740100	-0.14367900
C	1.30646400	-1.23819400	1.60089600
H	1.09642500	-3.35831300	1.30076000
C	0.32836200	-1.06874600	2.59713000
H	1.91447700	-0.38226200	1.33628600
H	-0.12715600	-1.94181900	3.06715700
H	0.35866700	-0.17681500	3.21171000
H	4.08730100	-3.77909100	-1.03418500
C	4.27035500	-3.53706000	1.13917900
H	2.89662900	-4.68387300	-0.08077800
O	3.31462000	-3.17311900	2.13993800
H	3.68165200	-2.29386200	2.60486300
H	3.62633900	-0.98197300	0.08633400
H	1.75331900	-2.83941200	-1.07554600
H	4.97248600	-2.70655200	0.99072200
C	5.09510000	1.12690800	3.09832600
H	5.84338700	1.60961700	2.46226400
H	4.26354100	1.82624100	3.24001000
H	5.52344700	0.88584500	4.07440500
C	4.54572300	-0.12308500	2.41759100

O	4.39793500	-0.07324300	1.16151400
O	4.25956300	-1.09944800	3.17603000
N	1.29181900	0.63114800	-1.91104400
C	1.13553000	2.03071400	-1.53186500
C	1.64447800	2.31341300	-0.08930900
N	0.51524900	2.44948100	0.83485300
C	0.31941000	0.01984000	-2.62915800
C	0.68359200	2.26670300	2.17323100
O	1.78309700	1.99553000	2.65733700
O	-0.69943400	0.59169500	-3.03473600
C	0.54728700	-1.42957300	-2.97911200
C	-0.50261100	2.42745300	3.10611600
C	-0.35513300	-2.43879700	-2.56729900
C	-0.13830800	-3.74742700	-3.02470300
C	0.92960300	-4.05237700	-3.87262000
C	1.81729400	-3.05283500	-4.26464000
C	1.62442800	-1.74703900	-3.81292800
C	-0.18807800	3.14529200	4.27150000
C	-1.11862100	3.33661000	5.28746200
C	-2.38702000	2.77350500	5.16354100
C	-2.70936100	2.03626300	4.02485700
C	-1.79306700	1.85377400	2.97304900
P	-1.64993700	-2.05578600	-1.28482400
P	-2.31573700	0.85025400	1.48769000
C	-2.22822500	-3.73420400	-0.73544900
C	-3.13407700	-1.50646900	-2.24519700
C	-2.78684800	2.20588600	0.31314400
C	-3.94474900	0.13560400	2.00084600
C	-5.18030900	0.74052700	1.72435500
C	-6.37360700	0.12059500	2.10462400
C	-6.34955200	-1.10594100	2.77030200
C	-5.12464000	-1.71672500	3.05140500
C	-3.93326900	-1.10482300	2.66205700
C	-3.11661100	-1.27690500	-3.62680200
C	-4.27879500	-0.87010900	-4.28752900
C	-5.46717100	-0.68835200	-3.57940600
C	-5.49107500	-0.91141100	-2.20027000
C	-4.33275600	-1.31461000	-1.53736800
C	-3.09940700	-4.51793600	-1.51226100
C	-3.51069900	-5.77590100	-1.07007800
C	-3.06639800	-6.26993800	0.15886600
C	-2.21134600	-5.49736800	0.94547300
C	-1.79907100	-4.23925900	0.50065700
C	-2.73863600	1.96456500	-1.06769700
C	-3.09669700	2.96244200	-1.97709600
C	-3.50965000	4.21380100	-1.51996900
C	-3.55828800	4.46830100	-0.14696000
C	-3.19685000	3.47552000	0.76481300
H	2.03350500	0.04880800	-1.50238600
H	0.06536500	2.23744100	-1.59739300
H	2.25912100	1.48872900	0.27643600
H	-0.35314200	2.83760900	0.48880300
H	-0.79937300	-4.54588500	-2.70744100
H	1.06829200	-5.07478700	-4.21353900
H	2.65891700	-3.28506300	-4.91105400
H	2.31503700	-0.96087600	-4.10308000
H	0.81945600	3.53469700	4.37060200
H	-0.84846000	3.90516100	6.17278000
H	-3.12629500	2.89721400	5.95039400
H	-3.69652400	1.59393300	3.95442500
H	-5.21523700	1.69401500	1.20769700
H	-7.32252500	0.60028100	1.87924100
H	-7.27889700	-1.58634500	3.06402200
H	-5.09630100	-2.67426200	3.56430200
H	-2.98411100	-1.59500600	2.86235000
H	-2.19563000	-1.39338000	-4.18524800
H	-4.25012500	-0.69419800	-5.35968300
H	-6.36874400	-0.37161100	-4.09716700
H	-6.40878400	-0.76758000	-1.63637000
H	-4.36457900	-1.48258200	-0.46428200
H	-3.46279600	-4.14149800	-2.46358600
H	-4.18280700	-6.36850200	-1.68511500
H	-3.39043300	-7.24844700	0.50301800
H	-1.86718800	-5.86914500	1.90704300
H	-1.14733700	-3.63257100	1.12221100
H	-2.39409600	1.01282100	-1.44816500
H	-3.03087100	2.75193100	-3.04008800
H	-3.78049100	4.99303400	-2.22728600
H	-3.86952000	5.44364100	0.21762100
H	-3.22965900	3.69274500	1.82768600
Pd	-0.80598800	-0.89130600	0.75061900
C	1.84158300	3.01872200	-2.53272200
H	1.52390200	2.79138600	-3.55378600
C	2.58106200	3.57663900	-0.11143700
H	2.90181800	3.80651600	0.90657000
C	1.47608100	5.93116900	-0.20116200
C	0.75247000	6.86610700	-0.95160500
C	0.36305300	6.56928800	-2.25900300
C	0.69193100	5.33409800	-2.83053800
C	1.42038900	4.40827600	-2.08868500
C	1.81334400	4.70643800	-0.77157400
H	1.78044900	6.16183300	0.81724900
H	0.49536900	7.82728200	-0.51396200
H	-0.19636200	7.29995200	-2.83756200
H	0.38507200	5.10009100	-3.84742900
C	5.07706700	3.08202500	-0.64923100
C	6.02137500	2.69044300	-1.60466400
C	5.62528400	2.39964300	-2.91122600
C	4.27727000	2.49123800	-3.27672700
C	3.33853000	2.88854700	-2.32866400
C	3.73971500	3.19305300	-1.01702100
H	5.38218300	3.28762100	0.37296300
H	7.06735300	2.60450000	-1.32338800
H	6.36483100	2.09426100	-3.64688300
H	3.96537000	2.25762400	-4.29243300
C	5.02356600	-4.77068800	1.55698300
H	4.40349400	-5.62903500	1.82267700
C	6.35420000	-4.86014400	1.61766500
H	6.94911700	-3.98129200	1.36259000
C	7.12429200	-6.08518800	2.02133600
H	7.75726300	-5.88388400	2.89636500
H	6.45626100	-6.91693000	2.27039400

H	7.79666900	-6.41772500	1.21826200

RSR-TS

b3lyp/6-31g(d)&LanL2DZ,
el. energy = -3883.348815 a.u.
im. frequency -80.60

C	2.53416400	1.84903600	-0.44460200
C	1.39097000	1.66874000	-1.25445700
H	3.27839200	1.06010800	-0.42286100
C	1.32313700	0.58184900	-2.16545000
H	0.59994800	2.41325200	-1.21921900
H	2.24401200	0.09632200	-2.48208400
H	0.52833000	0.56222400	-2.90726900
C	7.52851100	0.13171100	-0.00064300
H	8.06496700	-0.02981600	-0.93960900
H	7.38772200	-0.81441600	0.53012700
H	8.15032500	0.77805200	0.63184200
C	6.18826800	0.82995500	-0.25038200
O	6.14324600	1.61165700	-1.24384200
O	5.24587400	0.59257700	0.56521600
C	2.63874900	2.87188600	0.66131300
H	1.66839900	2.95872600	1.16112300
C	2.95962900	4.25225600	0.04846300
H	3.11457100	4.98072100	0.85123200
H	2.10598900	4.58865800	-0.55485200
C	4.20266400	4.10356100	-0.83179900
H	5.07978200	3.94751100	-0.19146700
O	3.97578900	2.91517400	-1.60699100
H	4.83462500	2.33826500	-1.51979700
O	3.58790000	2.48805300	1.63619100
H	4.26878200	1.89508900	1.22437500
N	-2.78364600	-0.22377100	-1.93646800
C	-3.56495900	0.27238300	-0.81146200
C	-3.05283200	1.66804900	-0.34410800
N	-2.26762900	1.58713300	0.88116500
C	-2.27726700	-1.49066700	-1.96955900
C	-1.30019600	2.51686200	1.13540000
O	-1.05864100	3.42896400	0.34133300
O	-2.52320300	-2.32587400	-1.10041300
C	-1.40430700	-1.81171300	-3.15324000
C	-0.56980800	2.45381900	2.45653100
C	-0.11620700	-2.37996900	-2.98283600
C	0.62837900	-2.66332700	-4.13606800
C	0.11644600	-2.42692800	-5.41569000
C	-1.15566500	-1.88483500	-5.57154700
C	-1.90720100	-1.57302800	-4.43735000
C	-0.55152900	3.67563900	3.14831300
C	0.12665000	3.81516000	4.35494400
C	0.83205100	2.72906400	4.87011900
C	0.84551800	1.51861900	4.17907000
C	0.14188300	1.34525000	2.97475400
P	0.64681000	-2.50662600	-1.28321300
P	0.21866900	-0.30297700	2.10318000
C	2.39150000	-3.06260200	-1.56903800
C	-0.03076200	-4.06585800	-0.55233100
C	-1.30894900	-1.10771300	2.78273600
C	1.60624200	-1.18134400	2.95097800
C	1.41866800	-2.27464900	3.81050700
C	2.52280100	-2.95874300	4.32866800
C	3.81929500	-2.56085400	3.99675400
C	4.01360500	-1.46849100	3.14716800
C	2.91578800	-0.78595600	2.62624200
C	-0.89130500	-4.93358000	-1.23694800
C	-1.31010100	-6.12518500	-0.64175300
C	-0.87780900	-6.46193900	0.64210800
C	-0.02261500	-5.59967900	1.33168400
C	0.39788700	-4.40878000	0.73957500
C	2.68465200	-4.32042700	-2.12877000
C	4.00601500	-4.73396300	-2.28938200
C	5.05597600	-3.90975900	-1.87200000
C	4.77740500	-2.67549600	-1.28727700
C	3.45338400	-2.25475500	-1.13838100
C	-2.00984200	-2.02585100	1.98718600
C	-3.15442100	-2.66221400	2.47573000
C	-3.61287400	-2.39033000	3.76459100
C	-2.92496600	-1.47441800	4.56550200
C	-1.78375900	-0.83544200	4.07975900
H	-2.50026000	0.42926600	-2.65429400
H	-3.44177100	-0.46781200	-0.01915500
H	-2.37978900	2.08917500	-1.09356600
H	-2.53113100	0.92663900	1.60143000
H	1.63100700	-3.06313700	-4.04195200
H	0.72220700	-2.66491900	-6.28569400
H	-1.56157600	-1.69981700	-6.56197300
H	-2.90407000	-1.15328900	-4.54630300
H	-1.06849900	4.52280200	2.70893000
H	0.12019800	4.76844300	4.87575700
H	1.38678600	2.82268900	5.79954600
H	1.42716100	0.69449800	4.57655000
H	0.41684800	-2.59769400	4.07509700
H	2.36469200	-3.80503300	4.99260100
H	4.67325400	-3.10095100	4.39743200
H	5.00794800	-1.13880400	2.86135200
H	3.09527000	0.07149400	1.98790900
H	-1.24660200	-4.67695500	-2.22893100
H	-1.97800700	-6.78941200	-1.18421700
H	-1.20591200	-7.39000800	1.10283800
H	0.31844000	-5.85027900	2.33257200
H	1.06566900	-3.74851100	1.28678900
H	1.87785300	-4.98476400	-2.42565700
H	4.21603300	-5.70577600	-2.72862600
H	6.08522700	-4.23788900	-1.99078500
H	5.57660300	-2.03207100	-0.93390400
H	3.25606400	-1.30348200	-0.65556800
H	-1.68414800	-2.23652900	0.97655400
H	-3.68172900	-3.36396300	1.83605300
H	-4.50464700	-2.88249500	4.14378500
H	-3.27851500	-1.25270300	5.56908700
H	-1.26400800	-0.12174100	4.71129600
Pd	0.69963200	-0.29024800	-0.30199800
C	-5.09985800	0.36920500	-1.13489200
H	-5.45659900	-0.59276800	-1.51260600

C	-4.27203400	2.65595800	-0.23220800
H	-3.92286700	3.62197400	0.13931000
C	-5.79659700	2.49992900	1.87043400
C	-6.75260600	1.76389400	2.58077100
C	-7.20708700	0.53906500	2.08868300
C	-6.70955100	0.03477700	0.88110000
C	-5.76601800	0.77073100	0.16982500
C	-5.30873300	2.00482600	0.66397500
H	-5.44186200	3.45357500	2.25428700
H	-7.14221100	2.14965700	3.51901900
H	-7.94996000	-0.02687700	2.64428500
H	-7.05909500	-0.92185000	0.49990100
C	-4.91453400	3.87936600	-2.43710500
C	-5.44124900	3.77898400	-3.73059000
C	-5.87946000	2.54705100	-4.21998400
C	-5.79579000	1.39983200	-3.42120000
C	-5.28107400	1.50025500	-2.13115400
C	-4.84173800	2.74165000	-1.63767900
H	-4.56664100	4.83744300	-2.05861900
H	-5.50780300	4.66530300	-4.35566100
H	-6.28812600	2.47698700	-5.22450500
H	-6.13822600	0.43978100	-3.80104500
C	4.43731500	5.27954700	-1.73938300
H	3.65483500	5.46220900	-2.47801500
C	5.50793400	6.07514500	-1.68449400
H	6.28222900	5.85978300	-0.94580700
C	5.75283200	7.26294600	-2.57091000
H	5.85449700	8.18616500	-1.98359600
H	6.68693900	7.14782700	-3.13760700
H	4.93670600	7.40557200	-3.28754600

SRS-TS

b3lyp/6-31g(d)&LanL2DZ,
el. energy = -3883.341745 a.u.
im. frequency -9.98

C	3.52876400	2.10225100	-0.66883700
C	2.93947700	1.41148100	0.53574900
C	3.84258600	3.58306200	-0.33660500
C	1.64176100	1.61334100	1.01121800
H	3.55967700	0.65094800	1.00013200
C	1.11461100	0.86193900	2.09193100
H	1.03927200	2.41094900	0.57904400
H	1.80754300	0.29841300	2.71679500
H	0.23667400	1.23494200	2.60376200
H	2.91323300	4.11058400	-0.07890600
C	4.81225600	3.64140600	0.84779200
H	4.26645000	4.06134700	-1.22658200
O	4.23277500	2.82647400	1.86324400
H	4.97615300	2.22562200	2.23357700
C	7.42843000	-0.69916500	2.41184600
H	7.01092200	-1.70896100	2.49733400
H	8.22033000	-0.74274700	1.65444700
H	7.85951000	-0.39497400	3.36824800
C	6.34127500	0.27933700	1.95615500
O	5.70729200	-0.03206000	0.89873700
O	6.16538600	1.31037000	2.65975300
N	-1.83676300	1.62207200	-1.61359200
C	-3.09148300	1.37301300	-0.91628400
C	-3.02732000	1.85623200	0.56051500
N	-2.79474000	0.74946900	1.48247600
C	-1.28201100	0.68642900	-2.43921100
C	-2.21009400	0.99533600	2.68887100
O	-1.92878800	2.14534700	3.03497000
O	-1.83664000	-0.38616000	-2.67591600
C	0.02773100	1.05668000	-3.08179100
C	-1.90681400	-0.15152200	3.63015900
C	1.15569700	0.19679900	-3.03594500
C	2.31239800	0.59552700	-3.72141000
C	2.35400100	1.78517100	-4.45515300
C	1.23922300	2.61625500	-4.50389900
C	0.08367500	2.25072400	-3.81066900
C	-2.20699800	0.14836300	4.96856700
C	-1.92443400	-0.74110700	6.00010300
C	-1.29648000	-1.94790000	5.70254700
C	-0.97001100	-2.25332200	4.38135400
C	-1.27091700	-1.38244500	3.31905700
P	1.22423400	-1.27927700	-1.89475900
P	-0.77214800	-1.88489400	1.58799700
C	2.93252100	-1.95203000	-2.11885800
C	0.27928900	-2.63500400	-2.72994000
C	-2.41267500	-2.43099300	0.91428800
C	0.13396300	-3.48071200	1.83505100
C	-0.49873600	-4.73376400	1.86231900
C	0.25150500	-5.90124900	2.02138100
C	1.63927400	-5.83412100	2.15879300
C	2.27925500	-4.59299300	2.13097000
C	1.53273800	-3.42634900	1.96352300
C	-0.26358600	-2.52601000	-4.01649100
C	-0.89356600	-3.62098900	-4.61180400
C	-0.98936100	-4.83582500	-3.93078000
C	-0.44831200	-4.95324700	-2.64867300
C	0.18276800	-3.86109400	-2.05282200
C	3.28027200	-2.70628200	-3.25642000
C	4.57489600	-3.19868300	-3.40771700
C	5.54327500	-2.94573700	-2.43040900
C	5.21125400	-2.20092400	-1.30073500
C	3.91029700	-1.71499300	-1.14598600
C	-2.62407600	-2.42838800	-0.47179600
C	-3.84122100	-2.85865600	-1.00557500
C	-4.86339900	-3.29621100	-0.16316100
C	-4.66677700	-3.29927500	1.22005000
C	-3.45250800	-2.86920000	1.75787900
H	-1.32558400	2.47326800	-1.42430300
H	-3.23934200	0.29289600	-0.96006500
H	-2.18075200	2.53321400	0.69782100
H	-3.16718600	-0.16787800	1.27287000
H	3.20778700	-0.01110000	-3.65989700
H	3.26854000	2.05935400	-4.97295200
H	1.26346300	3.54321600	-5.06981000
H	-0.79677100	2.88709500	-3.85319700
H	-2.65119800	1.11411000	5.18440600
H	-2.17626200	-0.48419200	7.02498600

H	-1.04999400	-2.65253200	6.49207400
H	-0.46808600	-3.19136400	4.17583800
H	-1.57608500	-4.80369400	1.75341900
H	-0.25204300	-6.86427800	2.03577100
H	2.22022100	-6.74440200	2.27905000
H	3.35950600	-4.52992100	2.22523900
H	2.04040800	-2.46629500	1.91637300
H	-0.20861800	-1.58429400	-4.55038500
H	-1.31067200	-3.52112000	-5.61054100
H	-1.48055800	-5.68608600	-4.39667300
H	-0.51550500	-5.89408600	-2.10900900
H	0.60705800	-3.96945200	-1.05821200
H	2.53945100	-2.91441300	-4.02185000
H	4.82746400	-3.78017000	-4.29088200
H	6.55385700	-3.32624800	-2.55620200
H	5.94033000	-1.94777200	-0.53822700
H	3.67966300	-1.13935000	-0.25952900
H	-1.85776700	-2.06611300	-1.14275500
H	-3.98105000	-2.83568700	-2.08211300
H	-5.81259400	-3.62376000	-0.57840000
H	-5.45975000	-3.63175300	1.88456600
H	-3.31667600	-2.87302500	2.83470100
Pd	0.75113200	-0.42220400	0.40803500
C	-4.33176000	2.03304100	-1.62095800
H	-4.34539300	1.75101000	-2.67707100
C	-4.31772900	2.69456900	0.88738900
H	-4.30584400	2.98117100	1.94076600
C	-6.54369000	1.42811400	1.34520600
C	-7.58804700	0.65453000	0.82444900
C	-7.60372900	0.31163300	-0.52878600
C	-6.57426600	0.73671400	-1.37719900
C	-5.54114200	1.51445400	-0.86191300
C	-5.52433800	1.86008000	0.50073800
H	-6.53148800	1.69595100	2.39914200
H	-8.39074300	0.32226800	1.47738900
H	-8.41870000	-0.28674900	-0.92732600
H	-6.58323800	0.46572200	-2.43037200
C	-4.14354400	5.22949600	0.32612800
C	-4.04636500	6.21371800	-0.66511400
C	-4.04049600	5.85735300	-2.01495600
C	-4.13228500	4.51126600	-2.38984000
C	-4.23806900	3.53223900	-1.40515600
C	-4.24561800	3.89167100	-0.04563900
H	-4.13954600	5.50649600	1.37747300
H	-3.97402000	7.25981900	-0.38032500
H	-3.96575200	6.62672100	-2.77875700
H	-4.13019000	4.23252400	-3.44130400
H	5.77364600	3.21435600	0.53280000
H	2.77753100	2.10578900	-1.47013000
O	4.66013700	1.42695300	-1.16661700
H	5.13407300	0.98536000	-0.41273600
C	5.02615100	5.04606300	1.35016400
H	4.15206300	5.51252900	1.80845100
C	6.17819400	5.71550900	1.26896300
H	7.04142700	5.21872400	0.82199000
C	6.40257000	7.11908800	1.75630800
H	5.49317200	7.54132100	2.19779300
H	7.19588300	7.15324500	2.51562500
H	6.72245600	7.78106600	0.93932800

RRS-TS

b3lyp/6-31g(d)&LanL2DZ,
el. energy = -3883.335529 a.u.
im. frequency -67.06

C	1.40929000	2.46947400	1.05265000
C	2.13539000	1.46718400	1.71483200
H	0.33826400	2.48582800	1.23196700
C	1.45282900	0.56032700	2.56948900
H	3.21866400	1.48728900	1.65340300
H	0.46134100	0.82555100	2.92376800
H	2.03263800	-0.06559000	3.24537300
C	6.36277300	3.71280700	3.05239700
H	6.32696000	4.42377400	3.88087100
H	7.13675000	4.01327400	2.33715600
H	6.64651500	2.72428700	3.43322900
C	5.00763600	3.61765100	2.34193900
O	4.05627100	4.29003800	2.82756500
O	4.96748500	2.85961300	1.32326800
C	1.91622200	3.32895200	-0.08985000
H	1.17744400	3.19708100	-0.89351800
C	1.97068000	4.84954800	0.27860000
H	1.66643900	5.45996100	-0.57847400
H	3.02501900	5.05999100	0.47965200
C	1.18225900	5.20691800	1.55048600
O	1.44928900	4.18716700	2.51093000
H	2.45614600	4.17346500	2.65255200
O	3.14133400	2.90424400	-0.61913300
H	3.84152900	2.97912100	0.09705200
N	-1.99774200	0.93220000	-2.00718900
C	-3.04923800	0.23899800	-1.27822200
C	-3.14644600	0.72078900	0.20434600
N	-2.59433200	-0.25272500	1.13671600
C	-0.84646700	0.29258700	-2.38184300
C	-2.00458000	0.16493300	2.29376700
O	-1.86694000	1.36140700	2.55619400
O	-0.68040000	-0.91098300	-2.18449500
C	0.20996300	1.11946100	-3.04381300
C	-1.58724000	-0.87957900	3.30787400
C	1.57821200	0.78010800	-2.88097900
C	2.53076200	1.59696200	-3.50613400
C	2.15634500	2.67958700	-4.30374100
C	0.80946500	2.98138000	-4.49078300
C	-0.15599100	2.20415400	-3.85432300
C	-2.08968900	-0.63563600	4.59514900
C	-1.77722500	-1.46643500	5.66739000
C	-0.92469900	-2.54888300	5.46321500
C	-0.39542200	-2.78752300	4.19477500
C	-0.71149200	-1.97340100	3.09369900
P	2.13902800	-0.59045500	-1.73893600
P	0.11203200	-2.29106600	1.44771400
C	3.99341700	-0.46781600	-1.80003300
C	1.93402700	-2.15445900	-2.71335600

C	-1.21566800	-3.23651100	0.55699900
C	1.34387400	-3.62507300	1.81674200
C	1.00587800	-4.98691500	1.87603300
C	1.98371000	-5.94862400	2.13735900
C	3.31098200	-5.56531700	2.34233600
C	3.65933000	-4.21451300	2.28152200
C	2.68346200	-3.25230000	2.01598500
C	1.42425400	-2.19901800	-4.01717100
C	1.33441400	-3.41172000	-4.70422000
C	1.75325000	-4.59822000	-4.09892200
C	2.26587500	-4.56408900	-2.80034900
C	2.35552700	-3.35207700	-2.11372400
C	4.75629800	-1.02717600	-2.84205800
C	6.14521400	-0.89289700	-2.85314100
C	6.79582000	-0.19377200	-1.83180600
C	6.05089500	0.36694200	-0.79453800
C	4.66286300	0.21886100	-0.77824800
C	-1.04519100	-3.52012900	-0.80660400
C	-2.00426400	-4.25627500	-1.50312700
C	-3.15746400	-4.70569000	-0.85685100
C	-3.34142900	-4.42692500	0.49805000
C	-2.37419300	-3.70581400	1.20395000
H	-2.03492400	1.94161400	-2.05038600
H	-2.77189300	-0.81549700	-1.31044400
H	-2.54995500	1.62394300	0.33809700
H	-2.80001600	-1.23560300	1.00374800
H	3.58413800	1.39751200	-3.35733400
H	2.92495200	3.28585700	-4.77457200
H	0.50999600	3.81318500	-5.12210200
H	-1.20886200	2.42079000	-4.01479900
H	-2.72023700	0.23511800	4.74336500
H	-2.18377200	-1.25887700	6.65306300
H	-0.65763400	-3.20422200	6.28782700
H	0.28378700	-3.62189600	4.06371000
H	-0.02081900	-5.29950100	1.71288000
H	1.70710300	-6.99896500	2.17690000
H	4.07085700	-6.31619800	2.54146800
H	4.69079800	-3.90732200	2.43046000
H	2.96135400	-2.20352600	1.94720900
H	1.08936000	-1.28652400	-4.49887200
H	0.93645900	-3.42651300	-5.71580900
H	1.68360900	-5.54087600	-4.63540900
H	2.59562100	-5.48009700	-2.31732600
H	2.75809800	-3.34243100	-1.10411400

H	4.26935100	-1.56837700	-3.64697200
H	6.71934100	-1.33260000	-3.66504700
H	7.87733600	-0.08452000	-1.85116800
H	6.51759900	0.94510600	-0.00259200
H	4.09980400	0.67602300	0.01983400
H	-0.17540100	-3.15265300	-1.33293200
H	-1.84889600	-4.46313000	-2.55821600
H	-3.90914200	-5.26738100	-1.40495600
H	-4.23534700	-4.77001800	1.01173000
H	-2.52472800	-3.51729400	2.26243900
Pd	1.29549900	-0.34556300	0.62725100
C	-4.45053900	0.40536800	-1.96284000
H	-4.38670200	0.11363200	-3.01462700
C	-4.63818500	1.09843200	0.53170900
H	-4.71973100	1.39855600	1.57927200
C	-6.29798100	-0.83596800	1.05612000
C	-7.01708400	-1.93563500	0.57206500
C	-6.92394600	-2.30145100	-0.77196700
C	-6.10950400	-1.57323600	-1.64753400
C	-5.40115300	-0.47462800	-1.16888300
C	-5.49536800	-0.10468700	0.18430200
H	-6.37066800	-0.55014500	2.10288300
H	-7.65279800	-2.50357600	1.24613400
H	-7.48712600	-3.15383600	-1.14244800
H	-6.03304200	-1.86111500	-2.69344300
C	-5.34165000	3.52608400	-0.08361500
C	-5.59926500	4.45858600	-1.09639400
C	-5.48979200	4.09017300	-2.43855900
C	-5.12195500	2.78389200	-2.78441300
C	-4.87436000	1.85287000	-1.77821700
C	-4.98478900	2.22448100	-0.42625800
H	-5.42289400	3.81412100	0.96168400
H	-5.88542400	5.47338000	-0.83420300
H	-5.69205800	4.81860000	-3.21909900
H	-5.03968600	2.49529600	-3.82998000
H	1.56218200	6.17037100	1.92325900
C	-0.30692800	5.32485900	1.35719500
H	-0.85271400	4.39509100	1.18994600
C	-0.97645300	6.47971800	1.37016600
H	-0.42589500	7.40279000	1.56268300
C	-2.45386500	6.62927900	1.14172200
H	-2.66084700	7.28951700	0.28783400
H	-2.93120100	5.66297300	0.94516600
H	-2.94609000	7.08185900	2.01360100

(S,S)-DACH-Ph [(S,S)-L1] Trost Ligand investigation

TS Pro-R-A
b3lyp/6-31g(d)&LanL2DZ,
el. energy = -3501.073492 a.u.
im. frequency -95.94

C	2.46017000	-1.77353900	-0.94155700
C	2.24461800	-0.73769000	0.13335800
O	1.37922100	-2.71049400	-1.09557600
C	3.79350900	-2.54499000	-0.72102700
C	1.78907900	-1.06250400	1.43673600
H	2.93933900	0.09231600	0.07640600

C	1.65234100	-0.02940400	2.36238900
H	1.43416400	-2.05660400	1.69143700
H	2.12267600	0.92974000	2.19891700
H	1.21387900	-0.21306300	3.33730300
H	3.59925500	-3.37994900	-0.03418700
C	4.95613200	-1.70985300	-0.14032800
H	4.08156200	-2.98802100	-1.68262600
O	5.07118100	-0.47079300	-0.83823200
H	5.06976900	0.23459400	-0.14320500
H	1.12283300	-3.08598800	-0.23059500
H	2.51925500	-1.25666900	-1.90045500
H	4.73085900	-1.51390700	0.91610800
C	6.27892600	0.90379400	2.83156900
H	6.44392200	0.70513900	3.89337000
H	6.68440600	1.88776700	2.57020300
H	6.82743300	0.16378600	2.23543300
C	4.78372300	0.84220500	2.47624200
O	3.98606200	0.48308900	3.37561000
O	4.48007400	1.15795800	1.27461200
N	-1.17694600	-2.01471700	-2.22465200
C	-2.37412700	-2.81026200	-1.96742200
C	-2.26670900	-3.60926800	-0.65239100
N	-2.15200400	-2.74931200	0.53043400
C	-1.23898900	-0.86726500	-2.94390400
C	-1.15891500	-2.87845800	1.44407600
O	-0.15829600	-3.58219600	1.24255900
O	-2.29558900	-0.30416700	-3.24588800
C	0.08685100	-0.37313200	-3.49241000
C	-1.37178800	-2.23106000	2.79963900
C	0.74341600	0.80851100	-3.08049100
C	1.90892100	1.19712200	-3.76005000
C	2.41310500	0.45001100	-4.82588300
C	1.76338300	-0.71465600	-5.22613100
C	0.60842900	-1.11892400	-4.55703300
C	-1.15846600	-3.11353100	3.86908800
C	-1.33537100	-2.71261600	5.19137900
C	-1.71611200	-1.40080500	5.46107800
C	-1.90114700	-0.50155100	4.40983300
C	-1.73591300	-0.88510300	3.06825200
P	0.23560900	1.69953300	-1.53815800
P	-1.80841000	0.41726900	1.73262900
C	1.47506500	3.06625300	-1.36876400
C	-1.26867500	2.68274600	-1.97476400
C	-3.48156100	0.13241400	0.99545900
C	-2.00368800	2.00815300	2.65356600
C	-3.24908800	2.57314500	2.97108400
C	-3.32046000	3.79606300	3.64199800
C	-2.15296900	4.46905100	4.00653000
C	-0.90867900	3.91838800	3.69152200
C	-0.83471200	2.70170700	3.01299300
C	-1.84110100	2.70142900	-3.25316000
C	-2.94865400	3.51260200	-3.51601100
C	-3.49237500	4.31357900	-2.51161200
C	-2.92547200	4.30200100	-1.23418500
C	-1.82337100	3.49139800	-0.96742500
C	1.37558600	4.23569100	-2.14632400
C	2.33552000	5.24073100	-2.03410800
C	3.40817100	5.09656200	-1.14810300
C	3.51353500	3.94302600	-0.37268900
C	2.54885400	2.93776400	-0.47771100
C	-3.68843500	0.42128200	-0.36063800
C	-4.93544600	0.20215900	-0.95057700
C	-5.98996900	-0.31083100	-0.19327300
C	-5.79430300	-0.60927500	1.15841700
C	-4.54815000	-0.39212000	1.75087800
H	-0.25419000	-2.35493800	-1.93105600
H	-3.21245400	-2.10969700	-1.89268300
H	-1.35320200	-4.21116600	-0.67010800
H	-2.98000000	-2.23046500	0.79868200
H	2.44218200	2.08513700	-3.44231200
H	3.31958000	0.77648600	-5.32746100
H	2.15325800	-1.31131600	-6.04608200
H	0.09678300	-2.02788700	-4.86126900
H	-0.84192600	-4.12642700	3.64241700
H	-1.16637700	-3.41802800	5.99973400
H	-1.85449700	-1.06538500	6.48515100
H	-2.16156400	0.52534300	4.64111200
H	-4.16625500	2.06663400	2.68936000
H	-4.29201300	4.22271100	3.87694400
H	-2.21209800	5.42099900	4.52706000
H	0.00607500	4.43744900	3.96328300
H	0.13645300	2.29105800	2.75371100
H	-1.44265900	2.07078900	-4.03836200
H	-3.38465200	3.51393900	-4.51148500
H	-4.35297200	4.94348100	-2.72110000
H	-3.34060100	4.92165200	-0.44388700
H	-1.38803600	3.49533000	0.02840400
H	0.54942000	4.36233000	-2.83886900
H	2.24494600	6.13801000	-2.64107900
H	4.15584200	5.88154800	-1.06789600
H	4.33989000	3.78859300	0.31414200
H	2.67344600	2.06309100	0.14651600
H	-2.87612300	0.79221100	-0.97175600
H	-5.06071900	0.41656200	-2.00729600
H	-6.95865100	-0.48668900	-0.65347500
H	-6.60936500	-1.01364800	1.75288200
H	-4.40389600	-0.63685700	2.79954200
Pd	0.26168700	0.26136300	0.44204600
C	6.25751200	-2.46999200	-0.21842800
C	6.91204200	-2.98044500	0.82798700
H	6.66527400	-2.58153100	-1.22512100
H	6.49084300	-2.84483000	1.82640700
C	8.20747400	-3.73928100	0.75623600
H	8.09968500	-4.75534800	1.16120000
H	8.99060600	-3.24776300	1.34977600
H	8.56676800	-3.82287400	-0.27533800
C	-3.48511500	-4.54011100	-0.46560400
C	-2.63190900	-3.75790000	-3.16175500
H	-3.34162500	-5.11973200	0.45397000
H	-4.38027900	-3.91783900	-0.31216800
H	-1.73756300	-4.38016500	-3.31108300
H	-2.74788500	-3.14254400	-4.06093600

C	-3.71656400	-5.46902500	-1.66399000
H	-4.60898000	-6.08219800	-1.48865000
H	-2.87005100	-6.16441200	-1.75593300
C	-3.85550600	-4.65878200	-2.95785100
H	-4.76405000	-4.04117700	-2.90764900
H	-3.97761600	-5.32578100	-3.81995600

TS Pro-R-B

b3lyp/6-31g(d)&LanL2DZ,
el. energy = -3501.070645 a.u.
im. frequency -12.10

C	-2.56936800	2.15284200	0.97495500
C	-2.07110600	1.52463200	-0.31106900
O	-2.86375200	1.17615700	1.97002500
C	-3.79819000	3.07649800	0.76725800
C	-2.29995800	0.18119600	-0.62043800
H	-1.87035500	2.21814200	-1.12543900
C	-1.76594800	-0.38533200	-1.78607400
H	-2.74771100	-0.47238300	0.11904600
H	-1.57223800	0.24112100	-2.65419900
H	-1.91391600	-1.43661000	-1.98569300
H	-4.02078800	3.47956500	1.76353600
C	-5.07206400	2.38448400	0.20662900
H	-3.52817300	3.92486600	0.12671800
O	-5.19023400	1.10211000	0.82283400
H	-5.37323400	0.35571900	0.13552600
H	-3.79811600	0.89741400	1.73867100
H	-1.77473700	2.78750800	1.37672100
H	-5.92158200	3.01118200	0.52688100
C	-5.45453000	-2.66845500	-2.29431700
H	-5.32490700	-2.75247900	-3.37749100
H	-6.47486000	-2.94472400	-2.01096900
H	-4.77095100	-3.37272200	-1.80288100
C	-5.11553900	-1.25182400	-1.80411100
O	-4.35684100	-0.54920400	-2.52624400
O	-5.60967200	-0.93736900	-0.67769800
N	-0.54079300	-0.25361100	2.95256600
C	-0.51750600	-1.62856300	3.45626200
C	-1.23763900	-2.62432500	2.52481700
N	-0.58647400	-2.75202400	1.21093100
C	0.53277200	0.55913600	3.07964000
C	-1.34150900	-3.13660700	0.13396400
O	-2.57013200	-3.12788800	0.17301800
O	1.66798700	0.17089800	3.38327000
C	0.27511900	2.04544200	2.93400500
C	-0.65739300	-3.65493500	-1.11917300
C	0.78084900	2.83393100	1.87549100
C	0.58883700	4.22336700	1.92670100
C	-0.07347400	4.82600500	2.99796400
C	-0.57531500	4.04238100	4.03438900
C	-0.40110800	2.65952900	3.99488600
C	-1.29442300	-4.78848200	-1.65148200
C	-0.85434800	-5.39325600	-2.82436100
C	0.22794500	-4.84663300	-3.50998900
C	0.85602500	-3.70603200	-3.01231900
C	0.44080400	-3.09403800	-1.81581300
P	1.48655900	2.01912700	0.36601400
P	1.34411600	-1.57814400	-1.22632500
C	1.62558800	3.37563100	-0.88968800
C	3.27842500	1.75630100	0.74645000
C	2.54405900	-2.34935300	-0.04503200
C	2.36317400	-1.08535400	-2.68771800
C	3.74575500	-1.30397900	-2.77750900
C	4.46115700	-0.86197300	-3.89451600
C	3.80675900	-0.20388500	-4.93580500
C	2.42912200	0.01814400	-4.85613400
C	1.71554300	-0.41040100	-3.73804600
C	3.82391600	1.93465300	2.02440000
C	5.19161600	1.74103200	2.23766300
C	6.02611500	1.37005000	1.18341800
C	5.48760800	1.18826600	-0.09314900
C	4.12410300	1.37798400	-0.31037500
C	2.59184900	4.39064500	-0.77009700
C	2.68164300	5.40157900	-1.72645000
C	1.81683800	5.40992700	-2.82423400
C	0.86633700	4.39884600	-2.96388400
C	0.77441000	3.38802800	-2.00379800
C	2.82263100	-1.72420800	1.17690400
C	3.70949100	-2.30427700	2.08839300
C	4.33335600	-3.51329600	1.78469900
C	4.05799500	-4.15104200	0.57053100
C	3.16398600	-3.58084000	-0.33467600
H	-1.43665400	0.16639400	2.66933600
H	0.53856200	-1.90580300	3.53913600
H	-2.24790600	-2.25897600	2.31955200
H	0.40395400	-2.96744400	1.20800000
H	0.94219500	4.84719900	1.11404400
H	-0.20556400	5.90433700	3.00922000
H	-1.10696800	4.49895900	4.86433900
H	-0.79645200	2.04081100	4.79515900
H	-2.16330900	-5.17339800	-1.12938000
H	-1.36563000	-6.27211400	-3.20621900
H	0.57961800	-5.29348400	-4.43587500
H	1.68069900	-3.28030900	-3.57269000
H	4.27020200	-1.81319200	-1.97624200
H	5.53240400	-1.03738000	-3.94786900
H	4.36487500	0.13676900	-5.80350300
H	1.91023800	0.53030800	-5.66168300
H	0.64686800	-0.22374800	-3.68161600
H	3.18513600	2.19748000	2.85820500
H	5.60081300	1.88264500	3.23438800
H	7.08933000	1.22284700	1.35358000
H	6.12785000	0.89934600	-0.92239800
H	3.72192500	1.24035200	-1.30980600
H	3.28091100	4.38622600	0.06900700
H	3.43143000	6.18047700	-1.61681400
H	1.89027200	6.19682000	-3.56994200
H	0.19724400	4.39040100	-3.81995600
H	0.04740600	2.59205200	-2.12795900
H	2.34116000	-0.79491700	1.44732400
H	3.89205000	-1.79917200	3.03173200
H	5.02335100	-3.96640300	2.49164900

H	4.53131300	-5.09980600	0.33202900
H	2.94198000	-4.09935300	-1.26226200
Pd	-0.03056900	0.28188100	-0.53050200
C	-5.10355000	2.30078200	-1.30253400
C	-5.70968000	3.20524300	-2.07799900
H	-4.64444900	1.42177500	-1.76070600
H	-6.22201100	4.05611000	-1.61953700
C	-5.76289900	3.12688100	-3.57914700
H	-6.80098600	3.14103900	-3.93968300
H	-5.29310600	2.20445500	-3.93569700
H	-5.25758500	3.98254000	-4.05148200
C	-1.34327100	-4.02383900	3.17439100
H	-1.91343200	-4.66774600	2.49624700
H	-0.33151700	-4.45072600	3.25167800
C	-1.14768700	-1.64398300	4.86986700
H	-0.56429600	-0.96781000	5.50613000
H	-2.16033100	-1.22119800	4.80131000
C	-1.98803700	-3.99597600	4.56429200
H	-2.01347500	-5.00968900	4.98267900
H	-3.03244600	-3.66483700	4.47791400
C	-1.22250900	-3.04400800	5.48890400
H	-0.20589800	-3.43027100	5.65371300
H	-1.70083200	-2.98884500	6.47453500

TS Pro-S-A

b3lyp/6-31g(d)&LanL2DZ,
el. energy = -3501.065696 a.u.
im. frequency -132.87

C	2.62374300	1.84898300	-0.39959800
C	2.08044700	0.55584400	-0.96519600
C	4.05620200	2.09632000	-0.95427900
C	1.35883000	0.57663300	-2.18097000
H	2.69503700	-0.31667900	-0.79418900
C	0.97096000	-0.63571200	-2.75074600
H	1.03978800	1.51563700	-2.62620100
H	1.38331500	-1.57512100	-2.41542700
H	0.34328000	-0.65319900	-3.63593200
H	4.08951400	1.77352900	-2.00218900
C	5.16930200	1.38818100	-0.14183500
H	4.26484000	3.17367900	-0.94673800
O	4.78728200	0.07439600	0.27399900
H	4.71297300	-0.51144500	-0.52356100
C	5.47115000	-1.54430900	-3.84474600
H	5.39683100	-1.99422600	-4.83861300
H	6.18911900	-2.09573000	-3.23016500
H	5.85053600	-0.52119400	-3.97083600
C	4.09254200	-1.49128500	-3.16295200
O	3.09130400	-1.42675400	-3.91911900
O	4.08434200	-1.49415500	-1.88704900
N	-0.14673900	2.27351900	2.00766700
C	-1.11054000	3.35105900	1.78384800
C	-1.15415400	3.81881800	0.31602400
N	-1.61728700	2.76640500	-0.60016500
C	-0.35137900	1.34209900	2.97413000
C	-1.21190300	2.77499500	-1.90517400
O	-0.27117100	3.48002000	-2.28019400
O	-1.43084800	1.16634600	3.54527300
C	0.88850800	0.58068800	3.40600600
C	-1.98070000	1.96351800	-2.93499800
C	1.26486600	-0.69013000	2.92312800
C	2.41746000	-1.29247700	3.45402100
C	3.17346200	-0.66532800	4.44463400
C	2.78640100	0.58267400	4.92873400
C	1.64844500	1.19667600	4.40824100
C	-2.09786900	2.63461700	-4.16340900
C	-2.75893200	2.06937500	-5.24997600
C	-3.30359900	0.79379600	-5.12820000
C	-3.17782400	0.10102300	-3.92407000
C	-2.52806300	0.65916500	-2.80949200
P	0.32483900	-1.50840900	1.54961800
P	-2.32219000	-0.37787500	-1.27407700
C	1.28897700	-3.06043100	1.22857300
C	-1.17582600	-2.21038900	2.38326900
C	-3.67934400	0.29153500	-0.20431400
C	-2.92832800	-2.05084700	-1.77480700
C	-4.25493600	-2.47741400	-1.60830300
C	-4.63428000	-3.77002100	-1.97842300
C	-3.69734800	-4.65113400	-2.52109900
C	-2.37341500	-4.23789900	-2.68792900
C	-1.99039700	-2.95080000	-2.31012700
C	-1.51412400	-1.95873700	3.71956500
C	-2.66193100	-2.53053800	4.27688600
C	-3.48055200	-3.36206000	3.51339100
C	-3.14822800	-3.62262100	2.18115200
C	-2.00765000	-3.05003100	1.62111400
C	1.10732700	-4.21363900	2.01482800
C	1.86868200	-5.35833200	1.77855400
C	2.82717500	-5.37111100	0.76105400
C	3.02014700	-4.23111300	-0.01816300
C	2.25296400	-3.08643900	0.21041700
C	-3.51131900	0.30446300	1.18827900
C	-4.50587000	0.82390900	2.02028500
C	-5.68408100	1.33088500	1.47075200
C	-5.86217300	1.32825100	0.08414400
C	-4.86572400	0.81913600	-0.75001800
H	0.80541400	2.33966300	1.63838000
H	-2.08902200	2.94788300	2.06543000
H	-0.14315000	4.07767500	-0.01121200
H	-2.49704800	2.31971100	-0.36880300
H	2.73454800	-2.25957400	3.08246600
H	4.06402500	-1.15397800	4.82944700
H	3.36855500	1.08128800	5.69872600
H	1.34445400	2.17358600	4.77481100
H	-1.64005900	3.61380200	-4.24861600
H	-2.83526700	2.61822700	-6.18422900
H	-3.81614400	0.32803400	-5.96550200
H	-3.58553500	-0.90115600	-3.85491600
H	-4.99387300	-1.80665600	-1.18282400
H	-5.66465000	-4.08675700	-1.83976200
H	-3.99486100	-5.65649000	-2.80639800
H	-1.63448100	-4.91858100	-3.10133300
H	-0.95365700	-2.64625500	-2.41902800

H	-0.90269400	-1.30229200	4.32573300
H	-2.91081500	-2.32129600	5.31388600
H	-4.37115000	-3.80508000	3.95139300
H	-3.77634600	-4.26846200	1.57365800
H	-1.75749100	-3.27090500	0.58780100
H	0.37253500	-4.22037500	2.81291500
H	1.71306100	-6.24080700	2.39399600
H	3.41980600	-6.26467900	0.58239800
H	3.76547100	-4.20159000	-0.80704900
H	2.44505600	-2.22901800	-0.42080700
H	-2.59831100	-0.06817300	1.63342700
H	-4.33493600	0.84078800	3.09191600
H	-6.45751700	1.73790100	2.11674700
H	-6.77340300	1.73037800	-0.35078700
H	-5.00759100	0.84023300	-1.82648800
Pd	0.00944500	-0.23808700	-0.56980900
H	5.30819600	1.94732300	0.79060700
H	1.98152100	2.67912400	-0.71282500
O	2.61307300	1.88284700	1.03197500
H	3.11828100	1.09772500	1.31914700
C	6.47133200	1.37776200	-0.90079500
C	7.56528400	2.05759200	-0.54922700
H	6.48182700	0.75684100	-1.79889900
H	7.53432000	2.66699400	0.35666000
C	8.86625700	2.05992300	-1.30076300
H	9.69050800	1.70257300	-0.66823800
H	9.13823500	3.07357900	-1.62650300
H	8.81852100	1.42023500	-2.18845100
C	-2.05821900	5.06272200	0.15019500
H	-3.10001600	4.76279100	0.33900200
H	-2.00206900	5.38427700	-0.89565000
C	-0.77520900	4.53084400	2.72783900
H	-0.81680900	4.15774000	3.75758600
H	0.26397900	4.83904800	2.54156600
C	-1.68481900	6.21263000	1.09261700
H	-2.37648800	7.05139900	0.94615000
H	-0.68168700	6.58496600	0.83983000
C	-1.70309000	5.73761000	2.54910000
H	-2.72900600	5.45962800	2.83138100
H	-1.39917200	6.54505500	3.22657400

TS Pro-R-A-new
b3lyp/6-31g(d)&LanL2DZ,
el. energy = -3501.075482 a.u.
im. frequency -26.58

C	-2.55900900	-1.50742500	-2.02195700
C	-1.89137200	-0.21119700	-1.63095000
C	-3.88298800	-1.29061400	-2.77556400
C	-1.06898200	0.48623400	-2.53465000
H	-2.35035000	0.35668100	-0.82526800
C	-0.38833300	1.63002900	-2.10255400
H	-0.84072800	0.03708600	-3.49867900
H	-3.68160800	-0.72496000	-3.69459800
C	-4.97468900	-0.57899100	-1.94552600
H	-4.25410700	-2.27986600	-3.06914500
C	-3.56635700	5.07712500	-1.39052200
H	-3.79043100	5.43325900	-2.39893800
H	-3.34315900	5.93087900	-0.73958600
H	-4.45003500	4.58016900	-0.97295800
C	-2.37970800	4.09993400	-1.39706600
O	-1.84812300	3.81964200	-2.49205400
O	-2.04676000	3.64393200	-0.24561300
N	-0.07675600	2.80997900	1.34925200
C	-0.70826600	2.65723000	2.65848100
C	-1.99497800	1.80787600	2.57282100
N	-1.71395400	0.39202100	2.24840900
C	1.25423000	2.74673600	1.17101500
C	-2.66476300	-0.37672100	1.66439900
O	-3.68489000	0.12718200	1.16816900
O	2.06250700	2.32138300	2.01254900
C	1.77622800	3.30970900	-0.13408200
C	-2.54882400	-1.88817100	1.69453600
C	2.54924800	2.55472100	-1.04730600
C	3.11058900	3.21073800	-2.15248500
C	2.92015800	4.57943100	-2.35673100
C	2.13843700	5.31193400	-1.46842400
C	1.57050100	4.67386500	-0.36574700
C	-3.74654600	-2.50570500	2.09579600
C	-3.87415400	-3.88854700	2.16107400
C	-2.79559600	-4.68838700	1.79369700
C	-1.60699100	-4.09451700	1.37331900
C	-1.44873600	-2.69753900	1.31825900
P	2.59425200	0.70196100	-0.93030600
P	0.20447100	-2.00129600	0.80059400
C	3.32363300	0.14661600	-2.54141100
C	3.98808900	0.28472000	0.21013200
C	0.97955300	-1.79486100	2.47224100
C	1.08803700	-3.46844000	0.08940900
C	2.29962200	-3.93192600	0.62357900
C	2.99027600	-4.98316400	0.01267900
C	2.48058000	-5.59011100	-1.13435500
C	1.27022000	-5.14185400	-1.66971300
C	0.58243900	-4.08881500	-1.06789000
C	4.67463100	1.24913500	0.95905400
C	5.74206500	0.87185900	1.77802500
C	6.13439900	-0.46445000	1.85850600
C	5.45403200	-1.43080400	1.11318100
C	4.38807900	-1.05924800	0.29518000
C	4.69720000	0.27991000	-2.81287800
C	5.22316200	-0.14079200	-4.03414200
C	4.38995500	-0.71088000	-5.00038500
C	3.02834900	-0.86088700	-4.73778500
C	2.50124200	-0.43598400	-3.51597100
C	1.66258100	-0.61518200	2.78892300
C	2.23133100	-0.43975000	4.05483100
C	2.13143800	-1.44645900	5.01264300
C	1.45091200	-2.63059200	4.70682400
C	0.87246700	-2.80266800	3.45118700
H	-0.70108700	3.18756700	0.59897300
H	0.01880400	2.17555500	3.32193400
H	-2.60336700	2.19778800	1.75227300

H	-0.97718200	-0.06864400	2.77082000
H	3.68583000	2.64946600	-2.87958100
H	3.36752300	5.05983400	-3.22242500
H	1.96024100	6.37051700	-1.63342300
H	0.95327400	5.23572500	0.32848600
H	-4.58996600	-1.87193700	2.34674400
H	-4.81292600	-4.33205400	2.47913600
H	-2.87364900	-5.77167700	1.82277900
H	-0.78678300	-4.73616600	1.07436300
H	2.71064900	-3.47518400	1.51701300
H	3.92692800	-5.32817100	0.44268500
H	3.01780000	-6.40805000	-1.60631700
H	0.85597400	-5.61225100	-2.55721700
H	-0.36527100	-3.77412400	-1.49439000
H	4.36214200	2.28494900	0.92694000
H	6.26562800	1.62997000	2.35432700
H	6.96580500	-0.75245100	2.49645900
H	5.75477300	-2.47403700	1.16526200
H	3.87474300	-1.81926400	-0.28685300
H	5.35827300	0.70891100	-2.06608000
H	6.28621500	-0.02727000	-4.22902400
H	4.80277000	-1.04172300	-5.94952900
H	2.37401900	-1.31224500	-5.47863200
H	1.44442000	-0.56794200	-3.30869900
H	1.75327100	0.19091600	2.07330800
H	2.74480600	0.49246400	4.26865200
H	2.57348100	-1.31302800	5.99655300
H	1.36256600	-3.41764500	5.45107100
H	0.33355600	-3.71986900	3.23562900
Pd	0.26832100	-0.15684000	-0.80687200
C	-6.24203200	-0.45552700	-2.75864500
C	-7.43954300	-0.89798300	-2.37216100
H	-6.12681300	0.03384300	-3.72763400
H	-7.51336500	-1.37717400	-1.39773500
C	-8.70156800	-0.78093100	-3.17882800
H	-9.46943900	-0.21109200	-2.63704000
H	-9.13630600	-1.76898500	-3.38594900
H	-8.52586900	-0.28158800	-4.13831100
C	-2.80171200	1.85042800	3.89048900
H	-2.22700300	1.32951400	4.67190900
H	-3.72749900	1.28381500	3.74151400
C	-1.03406700	4.07528100	3.18820800
H	-0.08819500	4.62038200	3.29353300
H	-1.61722000	4.58594500	2.41005000
C	-3.11015700	3.27670100	4.35902000
H	-3.66110200	3.24389700	5.30735600
H	-3.76539700	3.76935600	3.62755400
C	-1.81388500	4.07952500	4.50661600
H	-1.19920100	3.64114900	5.30707000
H	-2.02917800	5.11260000	4.80690400
H	0.33417000	2.11716200	-2.74564500
H	-0.75989700	2.20450300	-1.25981100
H	-4.62151100	0.44003700	-1.71571400
H	-2.78950400	-2.08059800	-1.11676000
O	-1.71494500	-2.28190200	-2.88911700
H	-0.80785900	-2.18033400	-2.55657700
O	-5.21553900	-1.28631200	-0.73679400
H	-4.77087300	-0.80002000	-0.01413600

TS Pro-S-A-new

b3lyp/6-31g(d)&LanL2DZ,

el. energy = -3501.077905 a.u.

im. frequency -93.01

C	-3.15701000	-0.97693800	-1.05014900
C	-2.12231400	0.12238100	-0.97060300
C	-4.43825400	-0.47924000	-1.76022200
C	-1.41194900	0.56291300	-2.10455700
H	-2.31013400	0.86131200	-0.19384300
C	-0.55174600	1.66279800	-2.00727200
H	-1.45181500	-0.00999600	-3.02987700
H	-0.57746200	2.31681700	-1.14895200
H	0.12317100	1.92043000	-2.81066400
H	-4.88498800	0.34062700	-1.18319000
C	-5.49618000	-1.59230100	-1.95065600
H	-4.16241700	-0.07172100	-2.74106300
O	-5.99143200	-2.06595400	-0.71225400
H	-5.22547200	-2.07261900	-0.10576100
C	-2.76048900	5.63837800	-2.11993500
H	-2.18197900	6.56722100	-2.05118100
H	-3.55058900	5.69542600	-1.36246900
H	-3.20818300	5.55971400	-3.11322600
C	-1.84620000	4.44038800	-1.82080500
O	-1.70899600	3.58259700	-2.73260600
O	-1.31136600	4.42891600	-0.66754400
N	0.53880400	3.37043600	1.00606000
C	0.03289400	3.55341400	2.36146100
C	-1.25562000	2.74459200	2.62948800
N	-0.98280700	1.29571200	2.68766400
C	1.66718600	2.70662500	0.68797900
C	-1.95550100	0.38268900	2.47255700
O	-3.03598800	0.67856100	1.93623800
O	2.28216200	1.95131700	1.45738600
C	2.21416300	3.02442000	-0.68678700
C	-1.79187800	-1.00563500	3.05345800
C	2.71318200	2.03584100	-1.56755600
C	3.27468900	2.44987300	-2.78420800
C	3.36266600	3.80094800	-3.12547600
C	2.86854700	4.76940800	-2.25720400
C	2.29364300	4.37584600	-1.05009100
C	-2.57908200	-1.20293000	4.20344200
C	-2.65082100	-2.44214600	4.82726500
C	-1.94271400	-3.51877500	4.29116000
C	-1.16614600	-3.33332500	3.15287400
C	-1.07095200	-2.08382800	2.51099100
P	2.46780300	0.22641300	-1.20917300
P	0.10782500	-1.93997100	1.07153500
C	2.76986500	-0.60335200	-2.84471400
C	4.01383900	-0.27044100	-0.31669900
C	1.67137000	-2.22600300	2.02476100
C	-0.17460900	-3.49593000	0.11757100
C	0.88693100	-4.08723200	-0.58866900
C	0.66658800	-5.19338500	-1.41015900

C	-0.61915300	-5.72050500	-1.54799000
C	-1.68257500	-5.13012300	-0.86300300
C	-1.46747100	-4.02406200	-0.03770400
C	4.85256200	0.66226600	0.30769400
C	6.02893000	0.24501700	0.93476200
C	6.38017500	-1.10494000	0.95280800
C	5.54540600	-2.04280500	0.34086300
C	4.37273500	-1.62807900	-0.28775400
C	4.05699800	-0.74139200	-3.39641400
C	4.24084400	-1.36894900	-4.62833900
C	3.14424000	-1.87878400	-5.32871300
C	1.86438700	-1.76127600	-4.78719700
C	1.68095800	-1.13048600	-3.55410500
C	2.33460400	-1.09591300	2.52655900
C	3.42629400	-1.24243700	3.38488300
C	3.87936600	-2.51365400	3.73762600
C	3.23786200	-3.64355100	3.22518500
C	2.13767700	-3.50264600	2.37898000
H	-0.02894700	3.81509400	0.25639400
H	0.81747500	3.21992300	3.05237800
H	-1.93951400	2.90765300	1.79222200
H	-0.15262100	0.99602700	3.18504400
H	3.63539600	1.71309900	-3.49159000
H	3.80643100	4.08615900	-4.07541000
H	2.91825700	5.82298300	-2.51661500
H	1.89647700	5.12361400	-0.37208900
H	-3.15137900	-0.36671000	4.59466700
H	-3.26230500	-2.56891300	5.71606400
H	-1.99403700	-4.49938700	4.75603700
H	-0.62382300	-4.18085700	2.74896400
H	1.89292500	-3.69103800	-0.49081200
H	1.50191600	-5.64209300	-1.94124200
H	-0.79076500	-6.58227700	-2.18728500
H	-2.68841300	-5.52765500	-0.96937100
H	-2.30718200	-3.55821300	0.46716400
H	4.58183900	1.71031200	0.32044700
H	6.67174400	0.98277300	1.40795000
H	7.29744200	-1.42569800	1.43960500
H	5.80586300	-3.09764600	0.35197500
H	3.74601800	-2.36571800	-0.78037600
H	4.92023900	-0.36379200	-2.85738200
H	5.24247700	-1.46286200	-5.03954700
H	3.28959700	-2.37058000	-6.28680600
H	1.00648700	-2.16520000	-5.31797500
H	0.68567900	-1.05819400	-3.12671400
H	2.02569600	-0.09688200	2.23135300
H	3.93155900	-0.35639800	3.75793700
H	4.73309000	-2.62571000	4.40084000
H	3.58937000	-4.63814100	3.48790200
H	1.64829100	-4.39189700	1.99471400
Pd	0.09313200	-0.19102000	-0.58875500
H	-4.99699900	-2.41849900	-2.49953500
H	-2.75580800	-1.82861400	-1.61482300
O	-3.47210800	-1.50008800	0.24911500
H	-3.52283900	-0.74671500	0.88306600
C	-6.63701700	-1.10254000	-2.80574800
C	-7.90592200	-1.01037500	-2.40358800
H	-6.36233900	-0.80901600	-3.82064900
H	-8.14276900	-1.31727600	-1.38694000
C	-9.04215200	-0.51913700	-3.25448200
H	-9.82014800	-1.28772600	-3.36346300
H	-8.70555400	-0.23423700	-4.25776200
H	-9.53021800	0.35474500	-2.80086100
C	-1.94582100	3.16041600	3.94874400
H	-1.30777500	2.84968100	4.79013900
H	-2.88438200	2.60088600	4.03386300
C	-0.22272400	5.06953400	2.54138500
H	0.73791100	5.58743000	2.43182300
H	-0.85701700	5.39731600	1.70691200
C	-2.20105900	4.66794900	4.04217400
H	-2.67227300	4.90477500	5.00419000
H	-2.90836300	4.97249300	3.25844500
C	-0.88667400	5.43631300	3.87238300
H	-0.21272900	5.19835900	4.70901800
H	-1.06219700	6.51848500	3.90938000

TS Pro-S-B-new

b3lyp/6-31g(d)&LanL2DZ,
el. energy = -3501.066887 a.u.
im. frequency -258.20

C	0.26857400	0.68373000	-2.65802500
C	-0.92405500	1.15817100	-2.03149800
C	-2.02547900	0.35334900	-1.84607200
H	-0.92600300	2.17624700	-1.65604400
H	-2.02322200	-0.63574300	-2.30594600
C	0.88071400	4.38560900	-4.83763100
H	0.07551300	5.07833100	-4.56372300
H	0.70467500	4.03484700	-5.85713600
H	1.82460900	4.93619800	-4.78025400
C	0.89059500	3.22053900	-3.84057300
O	1.40968400	3.43114200	-2.71209800
O	0.33827000	2.14651100	-4.24628700
N	2.35547500	2.37685300	-0.28498400
C	2.25646100	3.30260100	0.84224100
C	0.80666000	3.76136800	1.11223900
N	-0.07261300	2.67104200	1.57543800
C	3.14537600	1.27910100	-0.23389100
C	-1.37902800	2.64408700	1.22366000
O	-1.81808800	3.39934400	0.33767900
O	3.61708100	0.80783100	0.80858300
C	3.53303000	0.66936200	-1.56503100
C	-2.36990100	1.76966000	1.96767000
C	3.25318400	-0.67492300	-1.90839200
C	3.78486500	-1.18238700	-3.10248400
C	4.58113400	-0.39374100	-3.93615300
C	4.83731100	0.93285600	-3.60140200
C	4.30847400	1.45815500	-2.42217200
C	-3.55264400	2.45568800	2.29476800
C	-4.61472000	1.82021200	2.93087800
C	-4.51634500	0.46293300	3.22864500
C	-3.35638000	-0.23657200	2.89625900
C	-2.26253300	0.38973100	2.27370000

P	2.00186800	-1.64014300	-0.93196400
P	-0.76275300	-0.61209600	1.80756400
C	1.63789400	-3.11440000	-2.00197500
C	2.94995500	-2.45991300	0.42903600
C	0.35003200	-0.19604900	3.23334500
C	-1.28852200	-2.35260500	2.15604600
C	-1.06887000	-3.01121000	3.37538600
C	-1.46213600	-4.34167800	3.54268600
C	-2.08279400	-5.02984900	2.49888000
C	-2.30338500	-4.38496900	1.27905600
C	-1.90073100	-3.06069500	1.10713200
C	4.30529500	-2.20720700	0.67915500
C	4.96568600	-2.87243400	1.71567900
C	4.28407500	-3.79403200	2.51118200
C	2.93160900	-4.04902300	2.26954300
C	2.26867500	-3.38559700	1.23810700
C	2.51835400	-4.20719300	-2.08530800
C	2.22305100	-5.29823800	-2.90339600
C	1.04154700	-5.31989300	-3.64845700
C	0.15538100	-4.24513800	-3.56883400
C	0.45179000	-3.15382300	-2.74940900
C	1.73804300	-0.19367400	3.03499500
C	2.60598900	0.11136000	4.08687900
C	2.09912400	0.41621500	5.35001400
C	0.71665000	0.42742900	5.55828000
C	-0.15301900	0.12877000	4.50890400
H	2.01819400	2.71332900	-1.20329500
H	2.64529000	2.77409400	1.71904500
H	0.37274000	4.11354400	0.17320600
H	0.22684300	2.13193000	2.38020600
H	3.56505900	-2.20139600	-3.39974200
H	4.98165300	-0.81725000	-4.85316000
H	5.43540200	1.56155700	-4.25491100
H	4.49151200	2.49564700	-2.16025600
H	-3.63022900	3.50295100	2.02347500
H	-5.51429000	2.37825900	3.17313300
H	-5.33860200	-0.05727800	3.71242700
H	-3.30196200	-1.29563600	3.12167500
H	-0.58477200	-2.49022300	4.19493400
H	-1.28142000	-4.83913800	4.49202000
H	-2.38667100	-6.06463700	2.63167800
H	-2.77794600	-4.91564600	0.45811600
H	-2.04905800	-2.56956900	0.14843000
H	4.84016300	-1.47696200	0.08398400
H	6.01677900	-2.66531200	1.89897500
H	4.80150700	-4.30915400	3.31631200
H	2.38871900	-4.76109800	2.88533500

H	1.21735400	-3.59545200	1.06109000
H	3.43634200	-4.20692500	-1.50573600
H	2.91642600	-6.13349900	-2.95609700
H	0.81195000	-6.17170700	-4.28296400
H	-0.76894700	-4.25469100	-4.14038200
H	-0.24743200	-2.32625200	-2.67945800
H	2.15429000	-0.39457700	2.05728900
H	3.67529400	0.11894900	3.89907100
H	2.77390900	0.65620400	6.16761400
H	0.31301300	0.67464300	6.53674600
H	-1.22440500	0.15371000	4.68314000
Pd	-0.02922500	-0.36924900	-0.52478700
C	0.75465200	4.90117500	2.15560300
H	-0.28888800	5.21903900	2.26276900
H	1.06851400	4.49961500	3.13126400
C	3.15892800	4.52224700	0.53143900
H	4.18627700	4.15661500	0.41564000
H	2.85337800	4.92452600	-0.44465500
C	1.64833300	6.09125500	1.78895800
H	1.58574700	6.85660600	2.57246100
H	1.27987700	6.55473200	0.86309400
C	3.09475000	5.62857500	1.58960300
H	3.49522700	5.25529900	2.54374300
H	3.73329200	6.46824300	1.28840600
C	-3.37722900	0.81879800	-1.32579300
H	-3.49199100	0.49920500	-0.27792100
O	-3.54319000	2.22698000	-1.42474300
H	-3.00075300	2.68557900	-0.74184300
C	-4.51059600	0.15924300	-2.13312100
H	-4.44077900	0.48085700	-3.18057300
H	-4.37065700	-0.92903200	-2.10686600
C	-5.91979100	0.49453200	-1.59293800
H	-5.93717600	0.22432300	-0.51930900
C	-6.97435900	-0.31007300	-2.30611400
C	-7.69042400	-1.29109100	-1.75254800
H	-7.13115600	-0.03846900	-3.35116900
H	-7.52826300	-1.52693600	-0.69850400
C	-8.72969500	-2.11377600	-2.46032600
H	-9.71142100	-2.01519300	-1.97698900
H	-8.83651600	-1.81107800	-3.50750100
H	-8.47713600	-3.18322400	-2.44020100
O	-6.22981700	1.86875000	-1.76753500
H	-5.39354100	2.35172200	-1.61230400
H	1.21820300	1.14488800	-2.46745200
H	0.24025600	-0.14573100	-3.35325300

NMR spectra

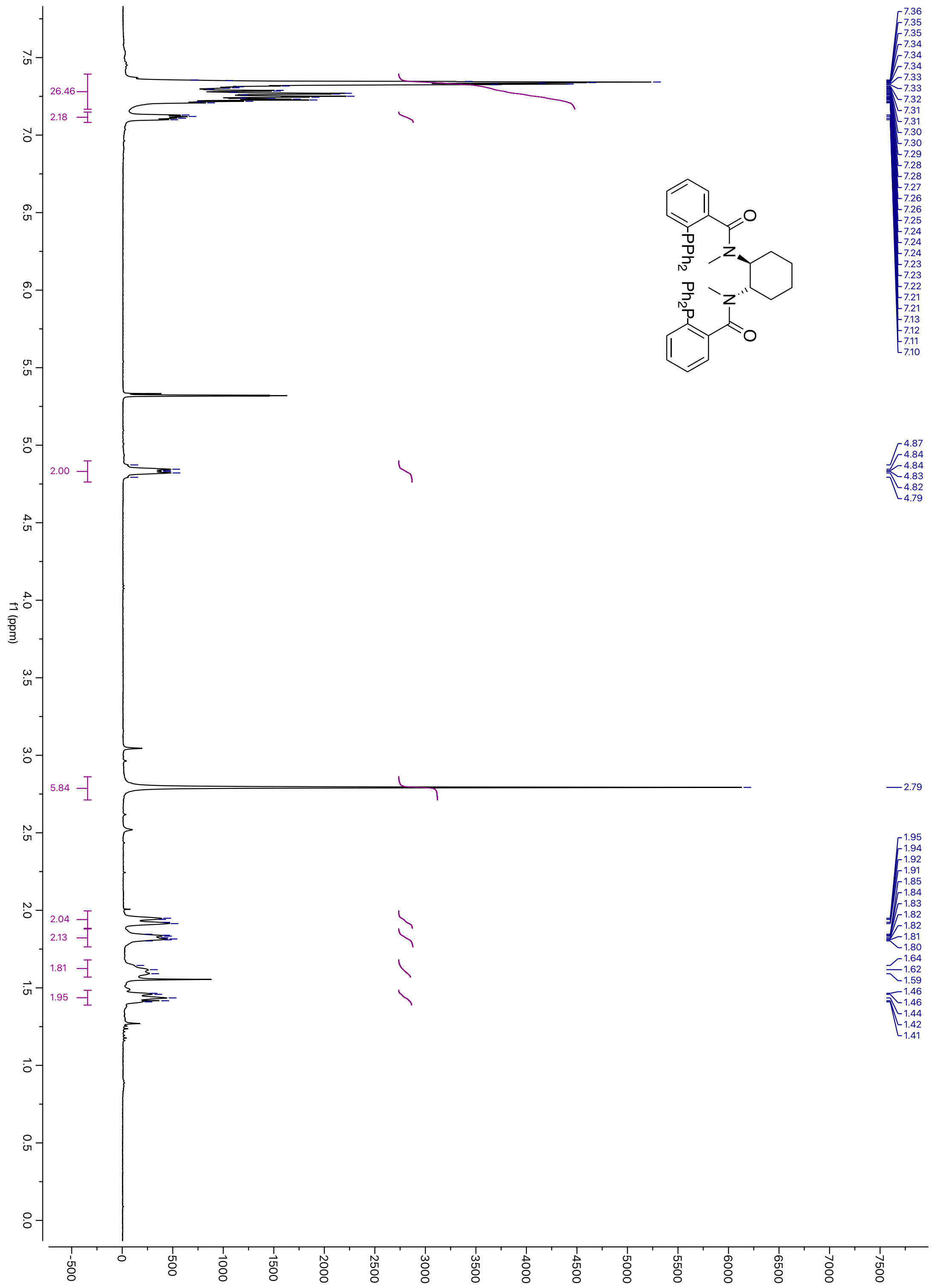

Figure S3: (*S*,*S*)-**L1**-Me [DACH-Me-Ph] ^{1}H-NMR (400 MHz, CD_2Cl_2)

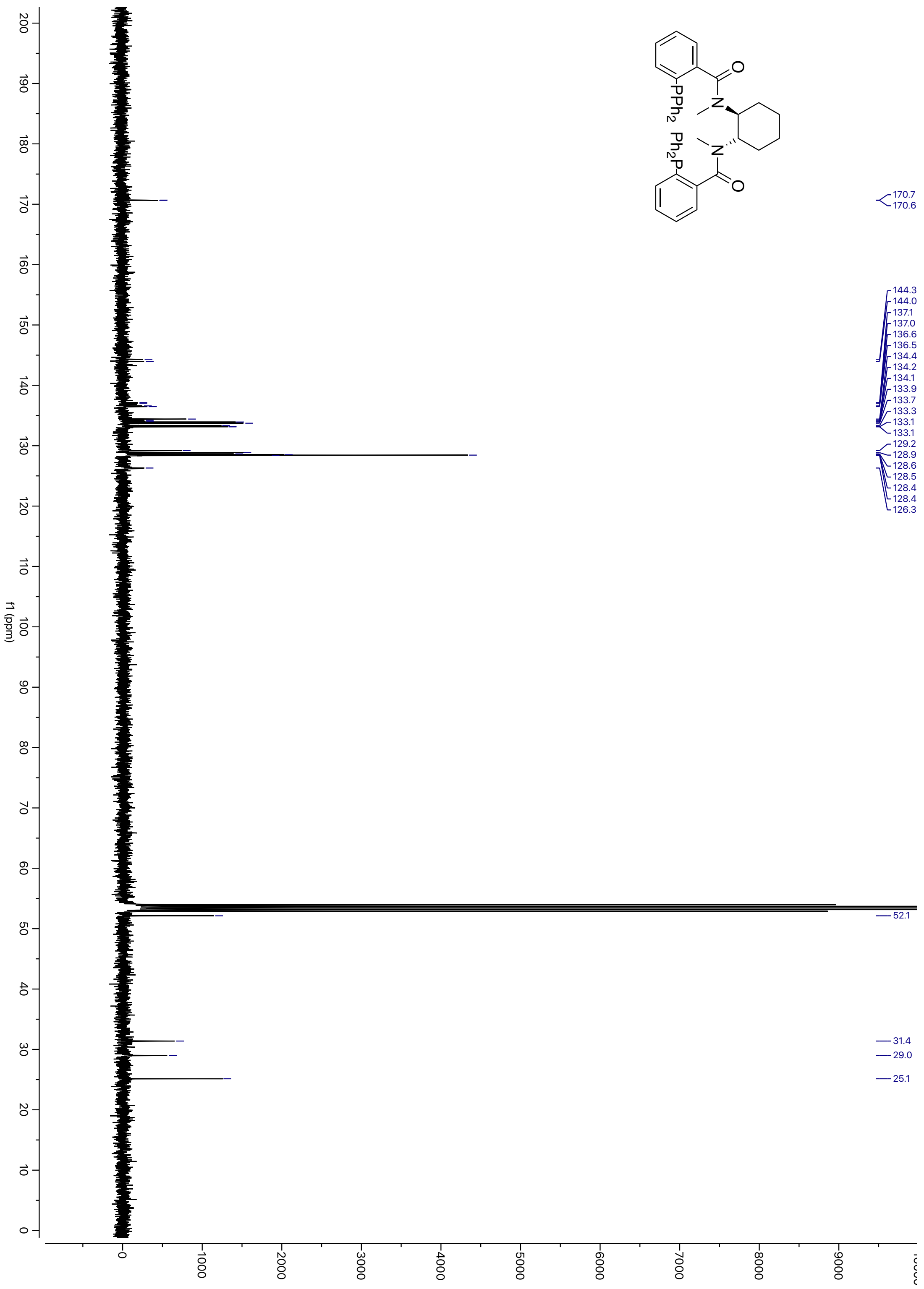

Figure S4: (*S*,*S*)-**L1**-Me [DACH-Me-Ph] ^{13}C-$^{1}\{H\}$-NMR (101 MHz, CD_2Cl_2)

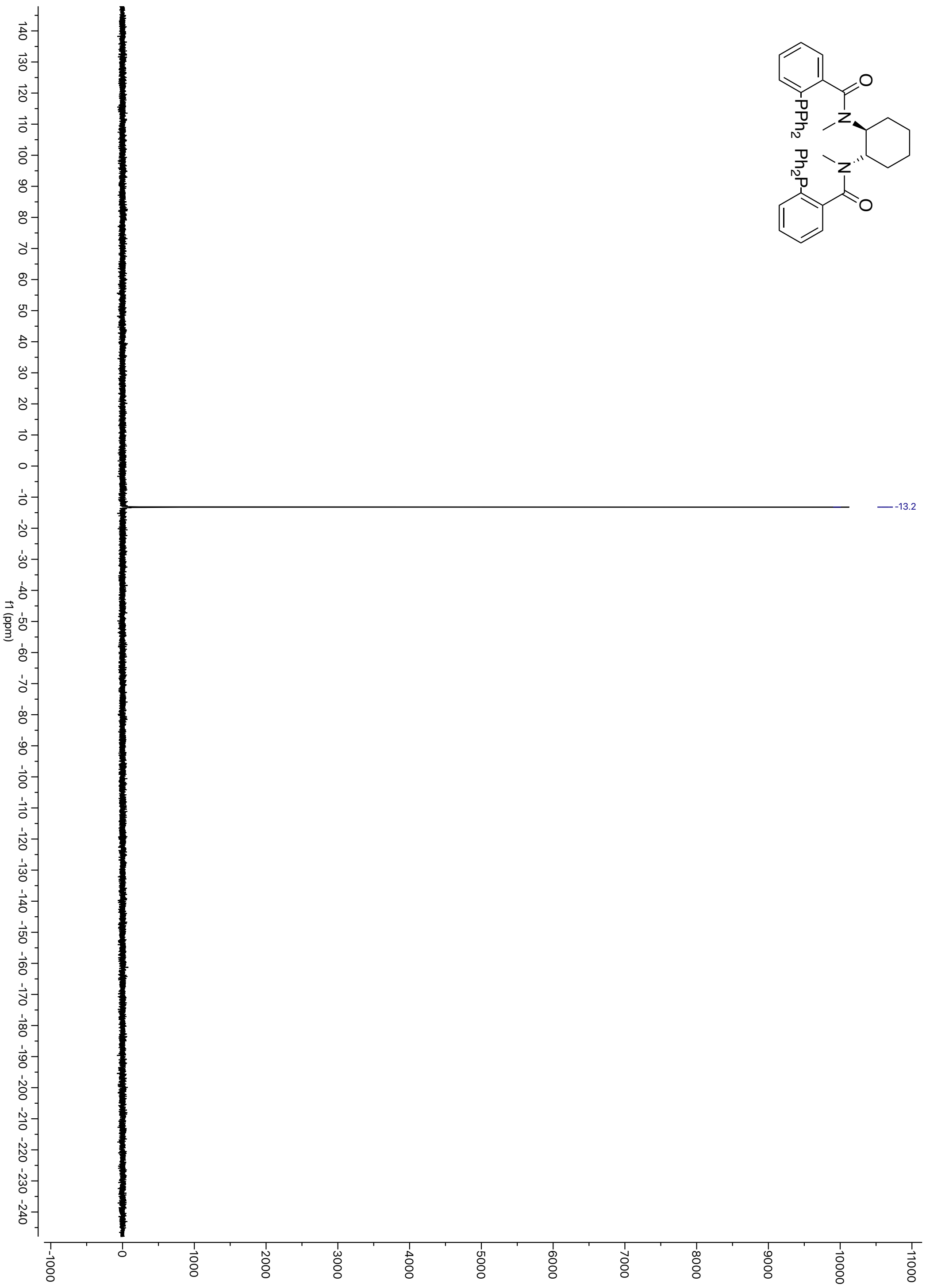

Figure S5: (*S*,*S*)-**L1**-Me [DACH-Me-Ph] ^{31}P-NMR (162 MHz, CD_2Cl_2)

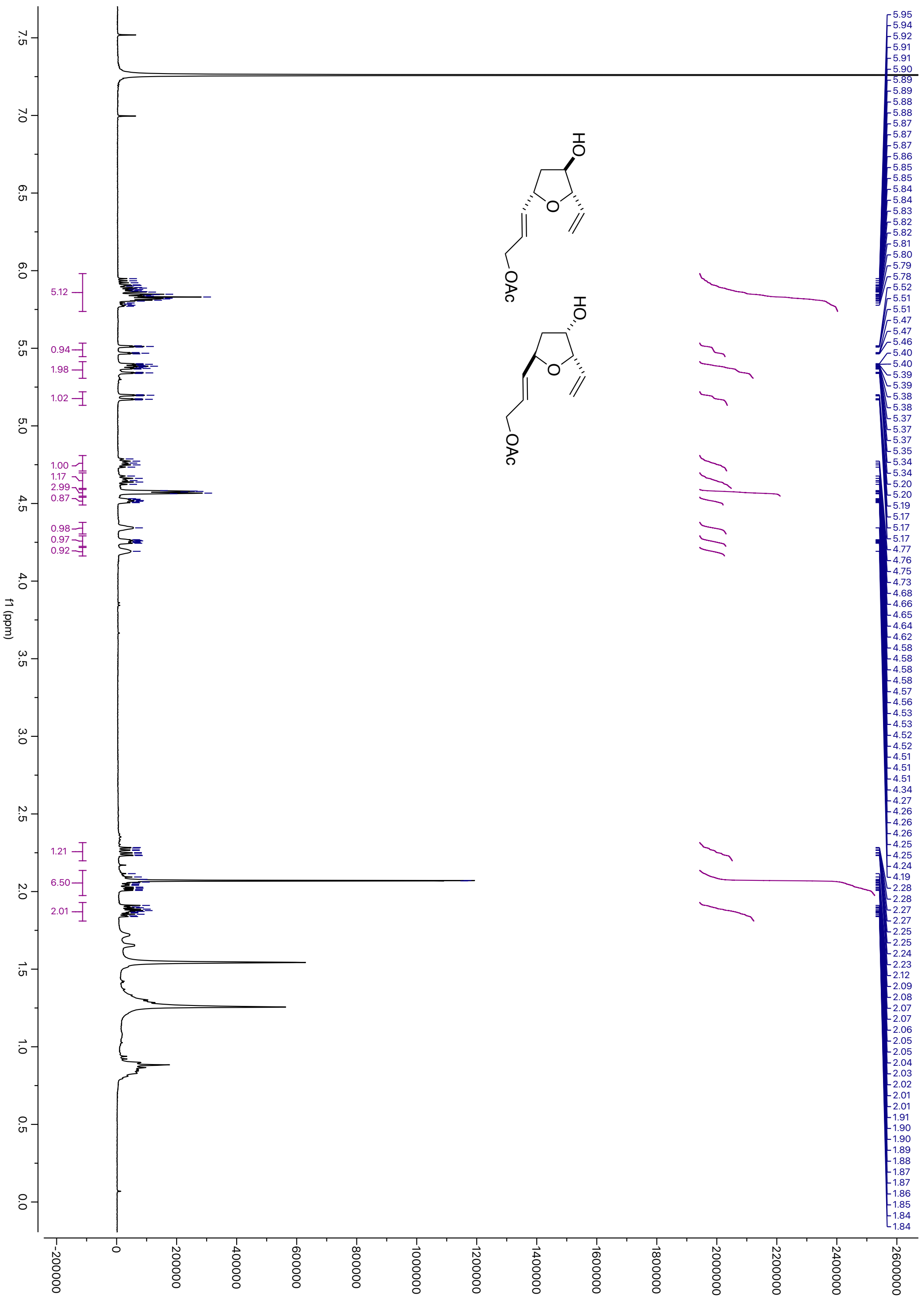

Figure S6: **2a-d** mix. of diastrereomers, ^{1}H-NMR (400 MHz, $CDCl_3$)

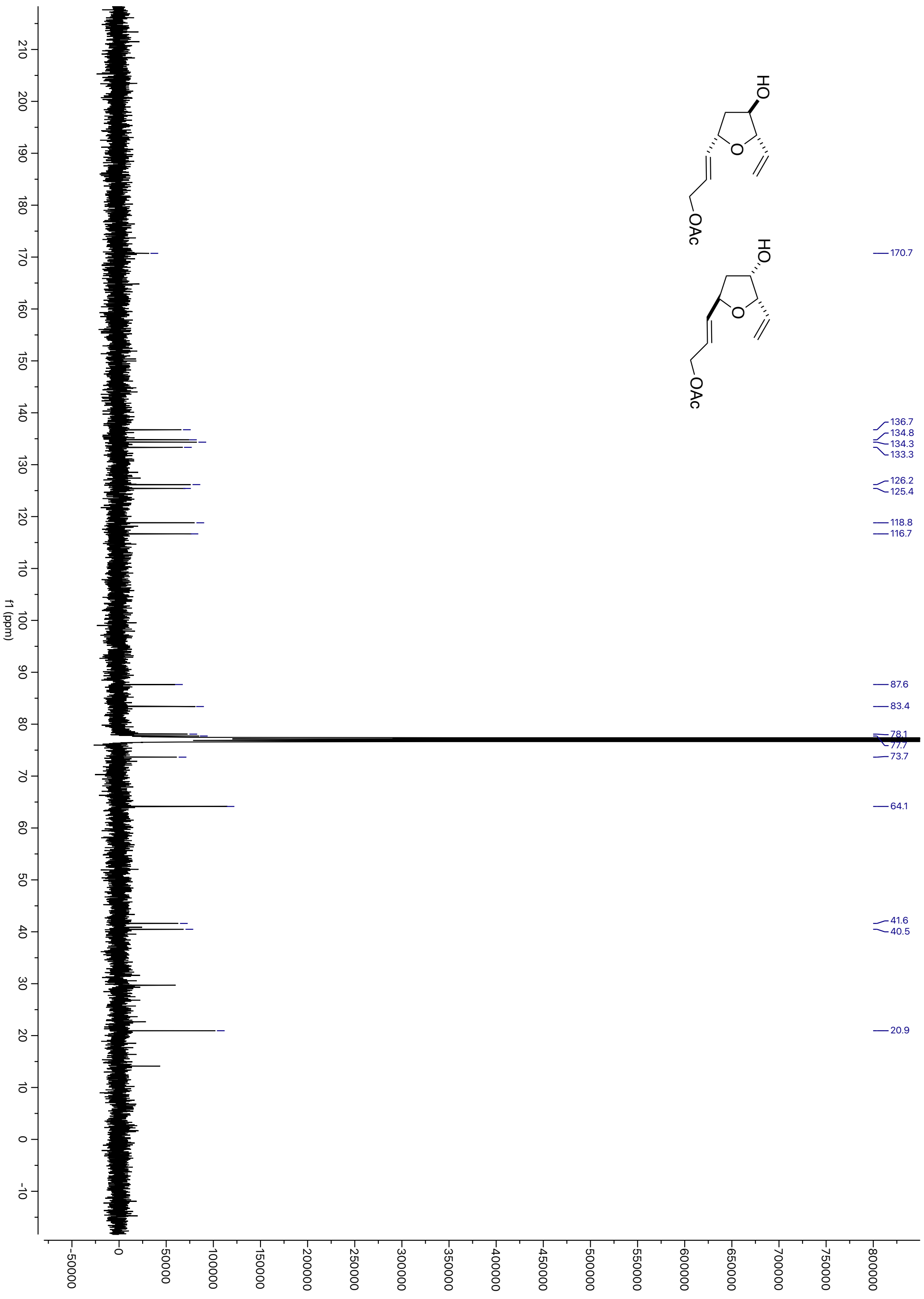

Figure S7: **2a-d** mix. of diastrereomers, ^{13}C-{^{1}H}-NMR (101 MHz, $CDCl_3$)

References

[1] Huang, D.; Liu, X.; Li, L.; Cai, Y.; Liu, W.; Shi, Y., Enantioselective Bromoaminocyclization of Allyl N-Tosylcarbamates Catalyzed by a Chiral Phosphine−$Sc(OTf)_3$ Complex, *J. Am. Chem. Soc.*, **2013**, *135*, 8101−8104.

[2] Valli, M.; Bruno, P.; Sbarbada, D.; Porta, A.; Vidari, G.; Zanoni, G., Stereodivergent Strategy for Neurofuran Synthesis via Palladium-Catalyzed Asymmetric Allylic Cyclization: Total Synthesis of 7-*epi*-ST-Δ^8-10-Neurofuran, *J. Org. Chem.*, **2013**, *78*, 5556−5567.

[3] Frisch, M. J.; Trucks, G. W.; Schlegel, H. B.; Scuseria, G. E.; Robb, M. A.; Cheeseman, J. R.; Scalmani, G.; Barone, V.; Mennucci, B.; Petersson, G. A.; Nakatsuji, H.; Caricato, M.; Li, X.; Hratchian, H. P.; Izmaylov, A. F.; Bloino, J.; Zheng, G.; Sonnenberg, J. L.; Hada, M.; Ehara, M.; Toyota, K.; Fukuda, R.; Hasegawa, J.; Ishida, M.; Nakajima, T.; Honda, Y.; Kitao, O.; Nakai, H.; Vreven, T.; Montgomery, J. A., Jr.; Peralta, J. E.; Ogliaro, F.; Bearpark, M.; Heyd, J. J.; Brothers, E.; Kudin, K. N.; Staroverov, V. N.; Keith, T.; Kobayashi, R.; Normand, J.; Raghavachari, K.; Rendell, A.; Burant, J. C.; Iyengar, S. S.; Tomasi, J.; Cossi, M.; Rega, N.; Millam, J. M.; Klene, M.; Knox, J. E.; Cross, J. B.; Bakken, V.; Adamo, C.; Jaramillo, J.; Gomperts, R.; Stratmann, R. E.; Yazyev, O.; Austin, A. J.; Cammi, R.; Pomelli, C.; Ochterski, J. W.; Martin, R. L.; Morokuma, K.; Zakrzewski, V. G.; Voth, G. A.; Salvador, P.; Dannenberg, J. J.; Dapprich, S.; Daniels, A. D.; Farkas, O.; Foresman, J. B.; Ortiz, J. V.; Cioslowski, J.; Fox, D. J. Gaussian 09, Revision B.01; Gaussian, Inc., Wallingford, CT, **2010**.

[4] a) Becke, A. D. Density-functional thermochemistry. III. The role of exact exchange. *J. Chem. Phys*. **1993**, 98, 5648-5652; b) Lee, C.; Yang, W.; Parr, R. G. Development of the Colle-Salvetti correlation-energy formula into a functional of the electron density. *Phys. Rev. B* **1988**, 37, 785-789.

[5] a) Hehre, W. J.; Ditchfield, R.; Pople, J. A. Self-Consistent Molecular-Orbital Methods. IX. An Extended Gaussian-Type Basis for Molecular-Orbital Studies of Organic Molecules. *J. Chem. Phys*. **1971**, 54, 724-728; b) Francl, M. M.; Pietro, W. J.; Hehre, W. J.; Binkley, J. S.; Gordon, M. S.; DeFrees, D. J.; Pople, J. A., Self-consistent molecular orbital methods. XXIII. A polarization-type basis set for second-row elements. *J. Chem. Phys*., **1982**, *77*, 3654-3665; c) Gordon, M. S.; Binkley, J. S.; Pople, J. A.; Pietro, W. J.; Hehre, W. J., Self-consistent molecular-orbital methods. 22. Small split-valence basis sets for second-row elements. *J. Am. Chem. Soc*., **1982**, *104*, 2797-2803; d) Hariharan, P. C.; Pople, J. A. The influence of polarization functions on molecular orbital hydrogenation energies. *Theor. Chim. Acta* **1973**, 28, 213-222; e) Hehre, W. J.; Ditchfield, R.; Pople, J. A. Self-Consistent Molecular Orbital Methods. XII. Further Extensions of Gaussian-Type Basis Sets for Use in Molecular Orbital Studies of Organic Molecules. *J. Chem. Phys*. **1972**, 56, 2257-2261.

[6] a) Hay, P. J., Wadt, W. R., Ab initio effective core potentials for molecular calculations. Potentials for K to Au including the outermost core orbitals, *J. Chem. Phys*., **1985**, *82*, 299-310.

[7] a) Gonzalez, C.; Schlegel, H. B. Reaction path following in mass-weighted internal coordinates. *J. Chem. Phys*. **1990**, 94, 5523-5527; b) Gonzalez, C.; Schlegel, H. B. An improved algorithm for reaction path following. *J. Chem. Phys.* **1989**, 90, 2154.

[8] Shubin, L., Steric effect: A quantitative description from density functional theory. *J. Chem. Phys.*, **2007**, *126*, 244103

[9] a) Tian, L.; Feiwu, C., Multiwfn: A Multifunctional Wavefunction Analyzer, *J. Comput. Chem*., **2012**, *33*, 580-592; b) Tian L., A comprehensive electron wavefunction analysis toolbox for chemists, Multiwfn, *J. Chem. Phys.*, **2024**, *161*, 082503.
